# Supplementary material for: Single-cell genomic variation induced by mutational processes in cancer
Source: Nature. 2022 Oct 26;612(7938):106–15. doi: 10.1038/s41586-022-05249-0 (PMC9712114; doi:10.1038/s41586-022-05249-0)
Supplement: Supplementary file 1 — SIGNALS validation and comparison and additional methods. [file 41586_2022_5249_MOESM1_ESM.pdf]

nature portfolio

https://doi.org/10.1038/s41586-022-05249-0

Supplementary information

Single-cell genomic variation induced by mutational processes in cancer

In the format provided by the authors and unedited

Supplementary Note: SIGNALS validation and comparison and additional methods

Allele and haplotype-specific copy number in scDNAseq 2

Input data 3

Algorithmic details 4

Validating haplotype-specific copy number calls with somatic mutations 7

Validation of phasing and parallel copy number evolution with SNVs 8

Consistency of haplotype-specific copy number calls with bulk whole-genome sequencing 9

Validation of phasing with long read data 11

Benchmarking against simulations 12

Influence of changing the transition probability 13

Accurate haplotype phasing in events present in single cells 13

Comparison with other methods 16

Running CHISEL and Alleloscope 17

Comparing rare cell populations 21

A note on GC bias and correction 22

SIGNALS with scRNAseq 24

Discussion of tools 26

Limitations of SIGNALS 26

Additional Methods 27

Breakpoint calling in single cells 27

Oxford nanopore long read sequencing 27

Allele bias in event rate calculation 29

References 30

Allele and haplotype-specific copy number in scDNAseq

Previously, we reported allele-specific copy number at the level of clones or clusters, groups of cells with similar total copy number profiles[1]. This was done by aggregating haplotype block counts within clusters and applying a hidden Markov model to infer the most probable state. In this study, we extended this approach to the single-cell level and also introduce the ability to identify "haplotype-specific copy number". We use the term haplotype-specific copy number to refer to phased allele-specific copy number[2]. With haplotype-specific copy number we can identify cells with the same total copy number but with different allelic combinations. We can also leverage haplotype-specific copy number to trace the history of complex genomic rearrangements.

First, we'll summarize the challenges of inferring haplotype-specific copy number in single cells and provide a descriptive overview of our approach. The majority of copy number analysis in single cells works by leveraging differences in read depth across the genome. Inference of allele-specific copy number requires an additional measure of allelic imbalance. In bulk sequencing, this is typically inferred from read count ratios of heterozygous SNPs. This information is very sparse in low coverage single cells, as typically at most one read will cover any individual SNP. In order to boost the signal, we can infer haplotype blocks from a paired normal sample using reference-based haplotype phasing, an approach that has also been employed in bulk tumor copy number tools and a recent single cell method[2,3]. The use of reference-based phasing increases the likelihood of multiple reads covering a particular allele in single cells, allowing computation of B-allele frequencies of individual alleles in single cells. Reference-based phasing however is only accurate over a range of a few 10's or 100's of kilobases, therefore we still have the challenge of phasing these haplotype blocks across the genome and reconstructing the homologous chromosomes, **Supplementary Note Fig.1a**. The fundamental challenge is therefore how to resolve the alleles in each block (what we will refer to as the "block alleles") into each homologous chromosome. To do this we can make use of allelic imbalance present in single cells or groups of cells, which in many cases unambiguously resolves how haplotype blocks phase into one of the two homologous chromosomes, **Supplementary Note Fig.1b**. For example, when a cell undergoes LOH it will lose representation of one of the homologs, in this case, the alleles with non-zero counts in each haplotype block must necessarily be phased together. In the case of a triploidy event, where one of the homologs is duplicated, in each haplotype block, we will observe one dominant allele (with BAF ~2/3) and one minor allele (with BAF ~1/3). In this case, by phasing all major alleles together and all minor alleles together we can recover the 2 homologs, **Supplementary Note Fig.1**. The same idea will work in any other scenario where cell(s) have allelic imbalance.

Once alleles have been phased into their respective homologs, we can then calculate the B-allele frequency in each bin. With this information, estimation of haplotype-specific copy number becomes relatively straightforward as the BAF is an effect a direct readout of allelic proportions, given that single cells are pure samples and are not influenced by normal contamination as in bulk sequencing. Of course in

real data, we may not want to rely on information from a single cell for phasing, and sampling noise due to the sparse coverage can be problematic. SIGNALS mitigates these issues by performing clustering to identify groups of cells with allelic imbalance and uses a hidden Markov model underpinned by overdispersed sampling distributions to account for variability in read counts. We describe these aspects in detail below.

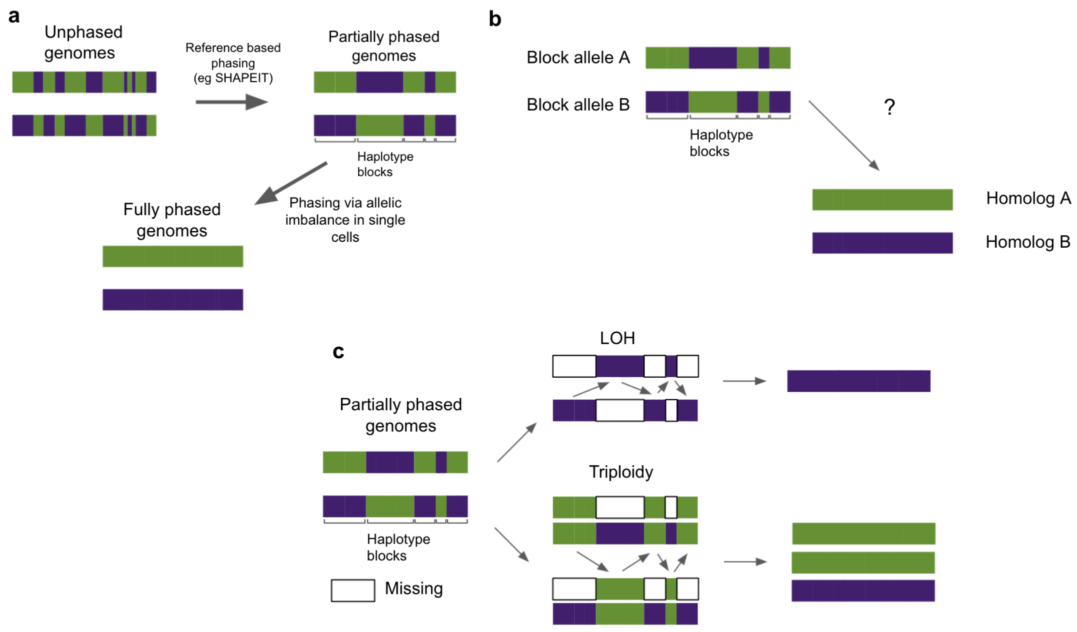

Supplementary Note Figure 1 Schematic overview of the inference problem and how it can be resolved using allelic imbalance in single cells

Input data

Before describing the algorithm in detail, we'll first describe the necessary inputs to SIGNALS. SIGNALS requires 2 inputs. The first input is total copy number estimates in bins across the genome. These should be integer states with chromosome, start and end positions. We used the single-cell HMMcopy implementation we first described in Laks *et al*[1]. This approach corrects read counts due to GC bias using a modal regression framework and removes bins with low mappability. In our experience, both of these are critical for the accurate estimation of total copy number. Our pipeline also contains cell quality and s-phase classifiers, allowing us to identify poor quality cells and cells undergoing replication. We remove such cells prior to inputting the data into the SIGNALS model.

The second input to SIGNALS is haplotype block counts per cell. This first requires estimating haplotype blocks. To do this we use a matched normal sample (or pseudobulk normal derived from normal cells in the same sequencing experiment) to identify heterozygous SNPs. We then use SHAPEIT - with the 1000 genomes as a reference - to estimate haplotype blocks[4]. Reference-based phasing is typically only accurate over a few 10's or 100's of kilobases. To incorporate this phasing uncertainty into our data and

identify adjacent SNPs where there is uncertainty over how the SNP should be phased, we implemented the approach to incorporate phasing uncertainty recommended in the SHAPEIT documentation. We sample from the graph outputted by SHAPEIT 100 times in order to identify alleles that are confidently phased together and conversely where there is ambiguity in the phasing assignment (see also Mcpherson et al[3]). Alleles that can be phased together confidently are assigned to the same block and given a label (`hap_label` column in our data files). We then genotype all heterozygous SNPs in our single-cell data and aggregate counts within phased alleles in each block in every cell.

Algorithmic details

In this section, we'll describe in detail the SIGNALS algorithm. We define the haplotype-specific state as follows: A|B where A and B are the copy number of the two haplotypes. The total copy number T is given by A + B, therefore both A and B <= T. Inferring the haplotype-specific state amounts to identifying the copy state of one of the two haplotypes. We define the "B allele frequency" as B / (B+A). In most cases, B will be the minor allele across the whole tumour population but our approach does not enforce this. We note that this is different from how this type of analysis is performed and the data is typically presented in bulk tumour genome sequencing. In bulk tumor methods, often both B/(A+B) and A/(A+B) are plotted, resulting in the characteristic split BAF plots in regions of allelic imbalance. As will become apparent, analyzing one of these values rather than both makes distinguishing haplotype-specific copy number intuitively easier. We note that we could in principle use mirrored BAF as is often done in bulk whole genome sequencing and define B as the minor allele in all cases. This is a simpler approach but does not allow for the identification of parallel copy number events and phasing alleles into homologous chromosomes.

As discussed above, the first challenge is to phase the alleles identified in the haplotype blocks into one of the two homologs (A, B). For the purposes of describing the algorithm, we'll denote the counts of each "block allele" as (C_{h1}, C_{h2}), and the counts of the phased alleles as (C_A, C_B). For each haplotype block in each cell we get the number of counts assigned to (C_{h1}, C_{h2}) respectively. Our challenge is to identify for each haplotype block how (C_{h1}, C_{h2}) relates to (A, B), that is we wish to know the phase P_i of each allele in each haplotype block, i, see **Supplementary Note Fig.1**b. This gives the counts of the phased alleles ie haplotypes, (C_A, C_B). To do this we note that cells will share copy number events and thus we can leverage information across cells to identify alleles that shift in frequency together. As a first approximation we first assign the B allele to be the minor allele across all cells:

$$x_i = \frac{\sum_{j=1}^{N} C_{h1,j}}{\sum_{j=1}^{N} C_{h1,j} + C_{h2,j}} \quad [1]$$

$$P_i = \begin{cases} A, & \text{if } x_i \geq 0.5 \\ B, & x_i < 0.5 \end{cases} \quad [2]$$

Where N is the total number of cells and j is an index over all cells. When a particular region of the genome is in a balanced state across all cells, distinguishing A and B is not possible. In this case, (A, B) will be assigned randomly due to stochastic fluctuations in read counts.

With this phasing assignment we then assign counts to each homolog:

$$(C_A, C_B) = \begin{cases} (C_{h1}, C_{h2}), & \text{if } P_i = A \\ (C_{h2}, C_{h1}), & P_i = B \end{cases} \quad [3]$$

After this initial phasing assignment we then merge the phased haplotype block counts that reside within the same bin and compute a BAF value for each bin in each cell:

$$BAF = \frac{C_B}{C_A + C_B}$$

With these values we then use a HMM to compute the optimal haplotype-specific state. We used a beta-binomial emission model and the Viterbi algorithm to assign the states. Given observed total copy number, T unobserved B-allele copy number B, B-allele counts C_B and total counts C_T the likelihood is given by

$$\mu = \frac{B}{T} + \epsilon$$

$$p(C_B|\mu, k, \pi, \rho) = BetaBinom(C_B|\mu, C_T, \rho)$$

Where ϵ is an error term included accounting for noise in the data, which we set to 0.01 in the first instance. This is particularly important in LOH states, where due to noise the BAF is not always exactly 0.0. ρ is the degree of overdispersion in read counts, which is inferred from the data using the maximum likelihood approach available in the VGAM package[5]. The case when $\rho \rightarrow 0.0$ is equivalent to a Binomial likelihood.

We used the following transition matrix with $\delta = 0.95$, favoring self-transitions.

$$T_{nm} = \begin{cases} (1-\delta), & \text{if } n \neq m \\ \delta, & \text{if } n = m \end{cases}$$

Following the above steps gives us our first haplotype-specific assignment. However, this first assignment can have some inaccuracies. Because our initial phasing uses the minor allele across all cells, if there are a

number of overlapping events in different cells at different proportions we sometimes find implausible results, where for example a cell will switch phase in the middle of a chromosome, see **Supplementary Note Fig.2** for a diagram showing how this can arise. A second issue arises when the majority of the tumor is diploid but there is a small clone with allelic imbalance. In this case, the phasing will be dominated by the diploid clone and the clone with allelic imbalance will contribute minimally to the overall phasing. To avoid this, we go through a second round of phasing and inference. We assume that the most accurate phasing should favor results that minimize the number of apparent switches in phasing. Secondly, we only use cells identified to have allelic imbalance for phasing which reduces the influence of cells in balanced states which are not informative for phasing. To do this, for each chromosome we cluster BAF values from step 1, and then identify the cluster with the largest amount of imbalance in each chromosome. We then define the B allele as the minor allele of cells within this cluster (using equations [1-3]). Clustering is performed using umap and hdbscan as described below. A key parameter that can be modified is the size (in number of cells) of the smallest cluster, the default we use is 10. All haplotype block alleles across all cells are then reassigned their phase relative to this cluster. Following this reassignment, we then rerun the HMM. Prior to running the HMM, we also take advantage of this 2 step process to infer ϵ and ρ directly from the data and assess statistical support for the Binomial vs BetaBinomial likelihood model. ϵ is computed from the average BAF of states assigned as homozygous, we compute Tarones z-score to assess statistical support for BetaBinomial model[6]. If we find support for a BetaBinomial model ($z>5$), ρ is then computed using maximum likelihood estimation. The HMM is then rerun with these input parameters and new phasing producing the final allele-specific assignment.

SIGNALS is available as an R package at https://shahcompbio.github.io/SIGNALS/. As well as the haplotype-specific copy number algorithm, SIGNALS includes a large number of functions for plotting copy number profiles and heatmaps, clustering cells, integrating with scRNAseq (see below) and performing QC. A number of tutorials accompany the package at the above URL describing this functionality.

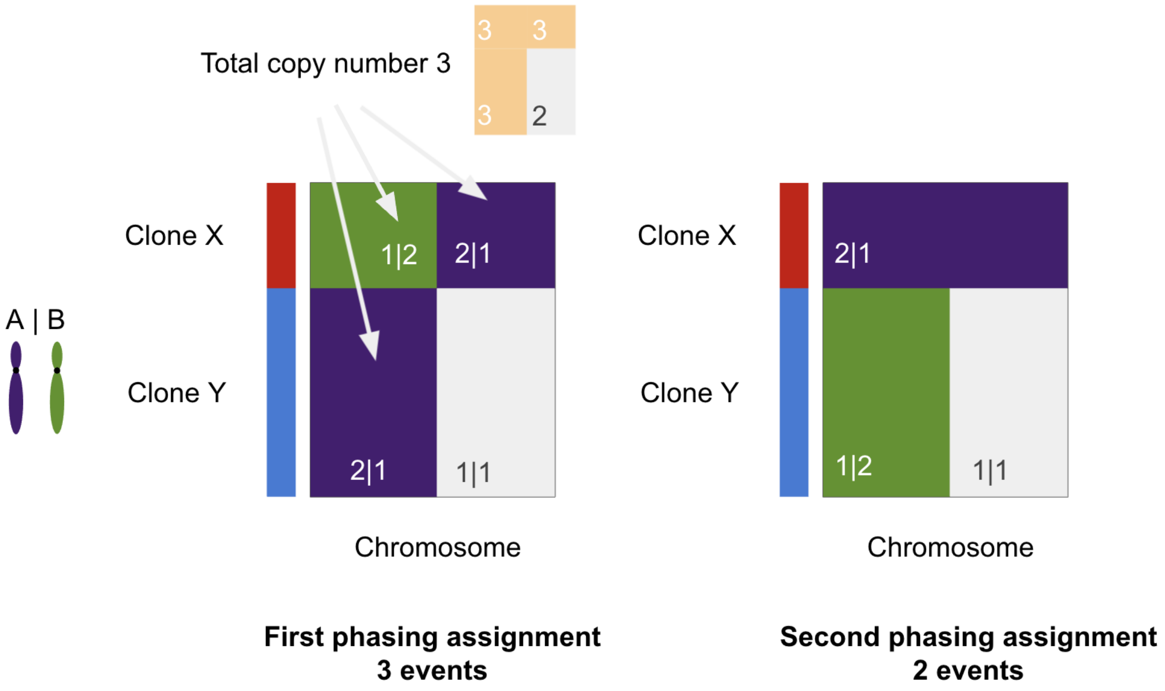

Supplementary Note Figure 2 Principle behind 2-stage phasing assignment

Validating haplotype-specific copy number calls with somatic mutations

To validate the accuracy of our haplotype-specific calls we took advantage of the distributions of somatic variants. We called somatic variants and genotyped them in single cells as described in the main methods section. We then mapped SNV's to haplotype-specific states in single cells using the function `snv_states` in SIGNALS. We could then take somatic mutations that were observed across different cells with the same haplotype-specific copy number and aggregate the counts, compute variant allele frequencies and plot their distributions. The variant allele frequencies (VAF) provide an orthogonal check that our inferences are correct. Modes in these distributions will reflect the underlying mutation copy number. For example, in balanced regions of the genome where A = B we would expect a mode at VAF of 0.5 (1 in every 2 genomes harboring the mutation). In regions of LOH, we would expect a mode at 1.0 (mutation present in all copies of the genome). Finally, in unbalanced but heterozygous regions (A≠B) we would expect modes that reflect mutations acquired pre and post the copy number alteration, for example, modes at 0.33 and 0.67 for 2|1 or 1|2 states. Inspecting these distributions across all our data we found that these were consistent with expectations, **Supplementary Note Fig.3**. Modes at low VAF that are evident in the balanced and LOH states reflect later acquired mutations some of which may be subclonal.

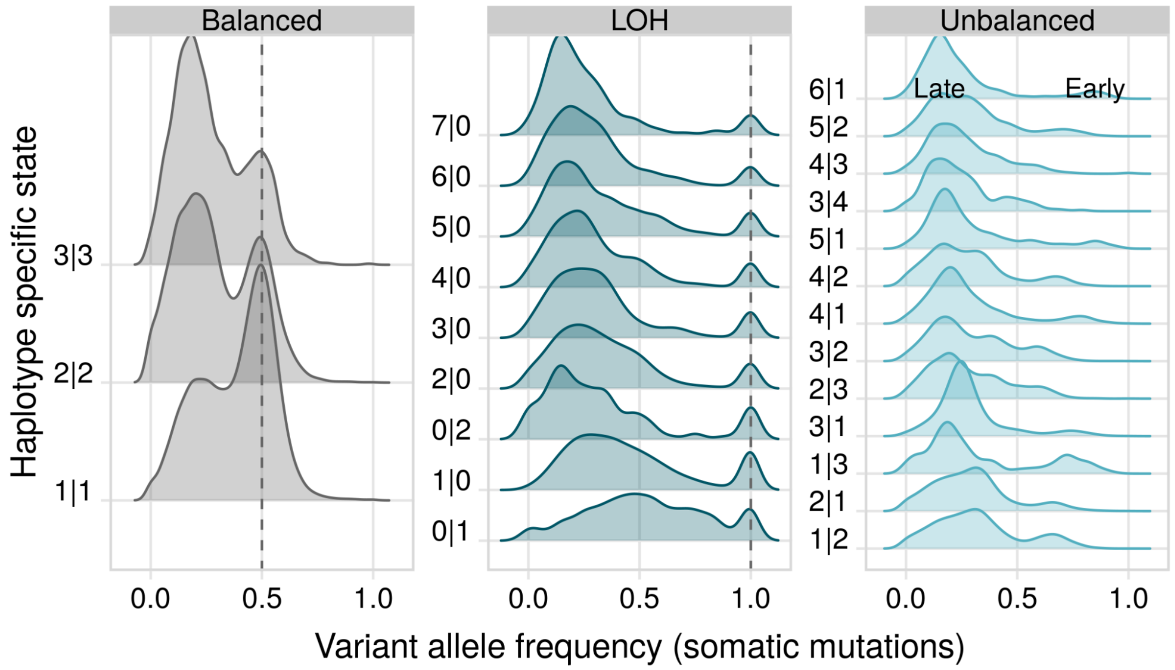

Supplementary Note Figure 3
VAF distributions per haplotype-specific state. Shown are distributions across the whole cohort of single-cell data.

Validation of phasing and parallel copy number evolution with SNVs

We can also take advantage of the VAF of mutations in different subpopulations with different haplotype-specific copy number calls to validate the haplotype phasing. We reasoned that ancestral mutations acquired when the chromosome was in a diploid state would originally be present in 1/2 copies, subsequent aneuploidies will then shift this mutation copy number up or down and influence the variant allele frequency of the somatic mutation. For example, for a parallel copy number event where 1 clone has a haplotype-specific copy number = 2|1 and a second clone has a haplotype-specific copy number = 1|2, the VAF of an SNV would be expected to flip between 1/3 and 2/3 in the 2 clones. Such inverting of the VAF of the same somatic point mutation provides a robust signature of the correctness of the phasing. This analysis is limited to cases where we can accurately infer ancestral mutations, and when we have sufficient depth to compute accurate VAFs. Within suitable datasets, we found numerous examples across multiple samples that support our haplotype-specific copy number inference, **Supplementary Note Fig.4**.

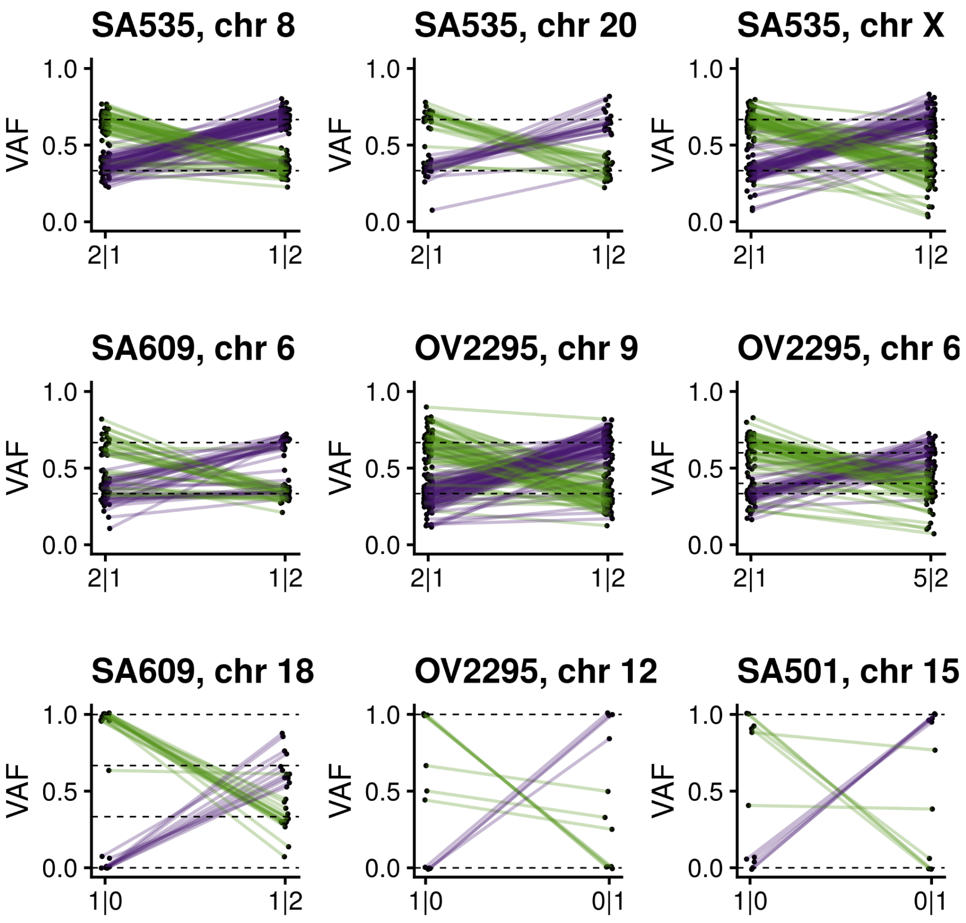

Supplementary Note Figure 4 VAF of somatic mutations as a function of haplotype-specific state
For each panel, we plot the VAF of each somatic mutation in 2 haplotype-specific states where the dominant allele switches between the 2 states. Each point is the VAF of a single SNV, lines connect the same SNV in the 2 states. If the line decreases from left to right it is colored green, if it increases it is colored purple. Dashed lines indicate where we expect the VAF to be based on the states. The title of each plot gives the dataset and chromosome.

Consistency of haplotype-specific copy number calls with bulk whole-genome sequencing

Another consistency check we can perform is to compare pseudobulk estimates of haplotype-specific copy number with those estimated from a matched bulk whole-genome sequencing experiment. We used Remixt to call allele-specific copy number in these data and generated consensus copy number profiles from all single-cell datasets using the `consensuscopynumber` function in SIGNALS. See **Supplementary Note Fig.5** contrasting remixt results and pseudobulk DLP data for SA1049 (whole-genome doubled) and SA1050 (baseline diploid). Visually we observe high concordance between the copy number calls. To assess this quantitatively across the whole cohort we computed the fraction of the genome called LOH and the average ploidy in both modalities as well as the correlation between mirrored BAF values. The fraction of the genome LOH and average ploidy were highly consistent (R = 0.91). Note that SIGNALS does not directly compute the per cell ploidy, this is done using HMMcopy but reliable estimates here are crucial for downstream accuracy in SIGNALS. Correlation of above 0.6 between mirrored BAF values was observed

for all but 1 dataset, **Supplementary Note Fig.6**. Bulk WGS data from this sample had low purity (<10%). We note that the bulk WGS and DLP are not exactly temporally matched. The bulk WGS data comes from primary tumor tissue while the single-cell data in most cases comes from PDX models. So discrepancies between the two modalities may be due to continued CNA evolution in the PDX models, rather than incorrect calls.

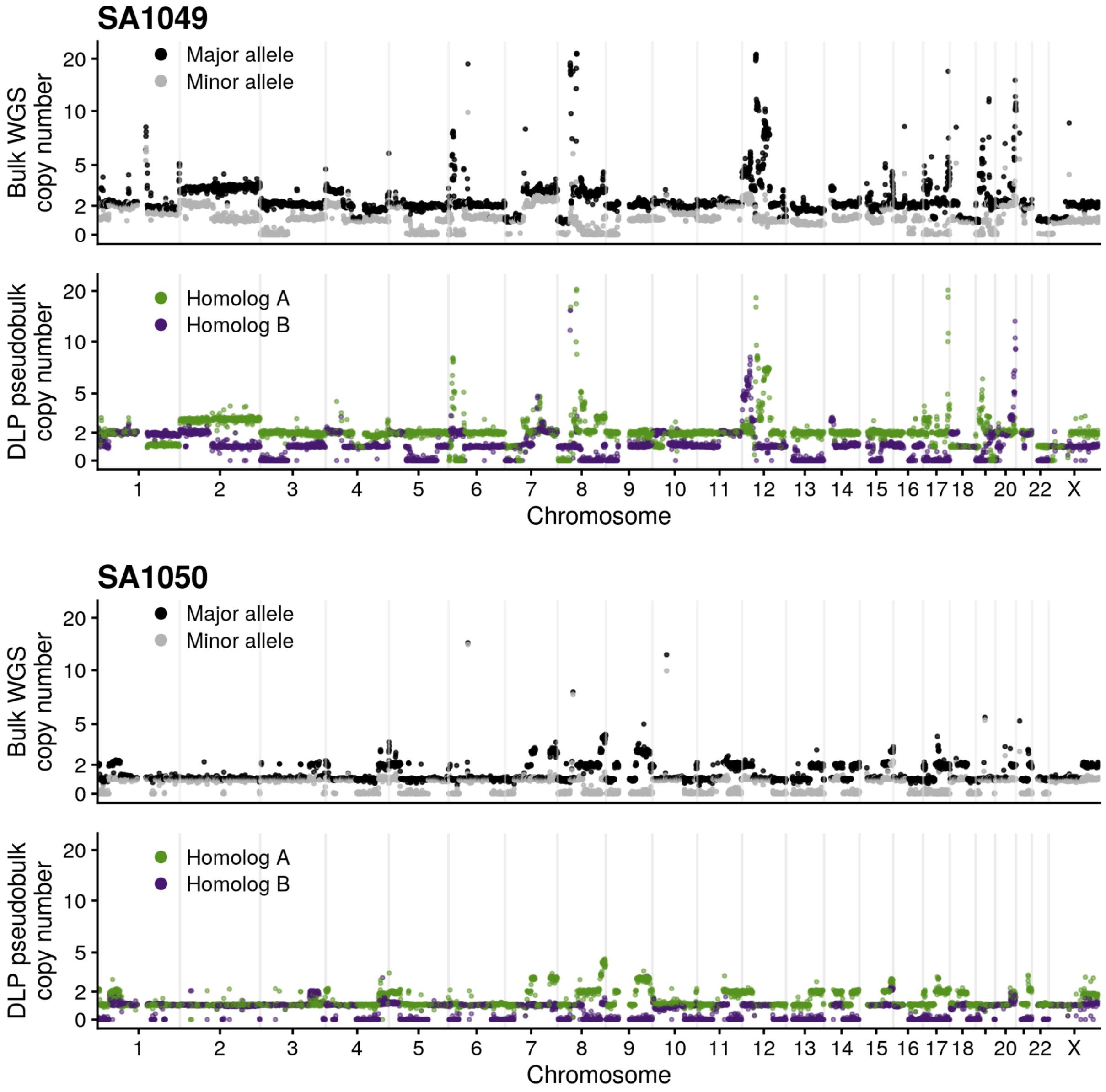

Supplementary Note Figure 5
Bulk WGS derived copy number versus single-cell pseudobulk derived consensus copy number for 2 datasets SA1049 and SA1050

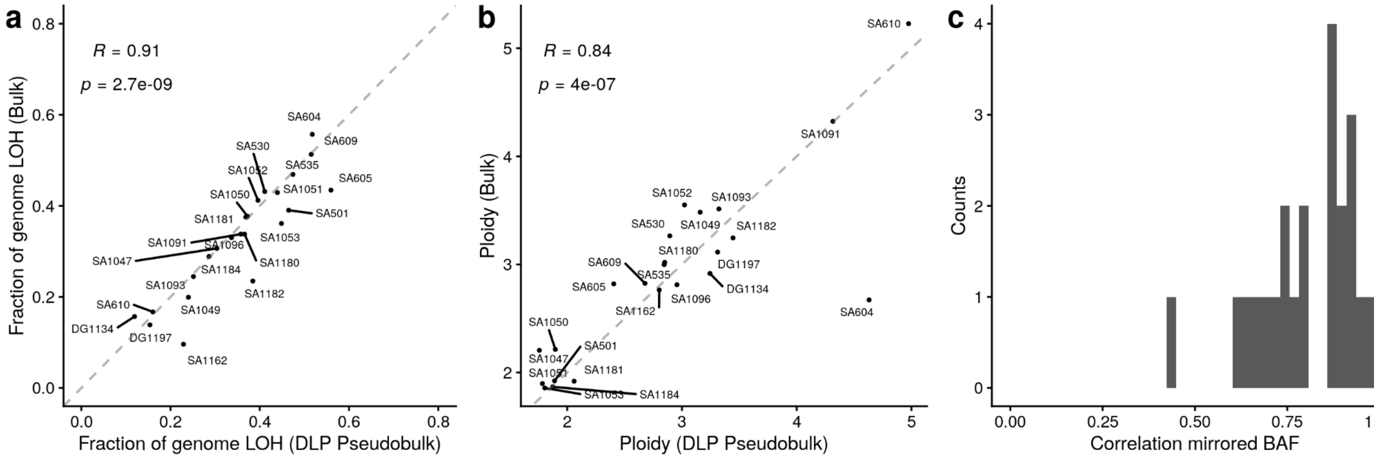

Supplementary Note Figure 6 Comparison of bulk WGS copy number calls with pseudobulk single cell copy number calls
a) Fraction of genome LOH in the modalities, dashed line indicates y = x. **b)** inferred ploidy in the 2 modalities. In **a)** and **b)** upper left annotations indicate the correlation coefficient (R) and p-value (p) derived from a linear regression using the lm function in R
c histogram of the correlation between mirrored BAF

Validation of phasing with long read data

We also generated long read sequencing data using oxford nanopore technologies (ONT) from the wild type hTERT cell line. We ran the PEPPER-Margin-DeepVariant pipeline[7] to phase heterozygous SNPs. Using the ONT data as ground truth, we contrasted the phasing produced from SIGNALS with random phasing and the phasing from SHAPEIT (used as input to SIGNALS). We used standard metrics to assess the quality of phasing; the switch error rate and the blockwise hamming distance. Phasing information was encoded in a vcf in the standard format (1|0 for phased variants vs 1/0 for unphased variants) and code provided by the WhatsHap algorithm was used to compute these metrics[8]. We found that SIGNALS had lower values for both these metrics, indicating improved phasing, **Supplementary Note Fig.7**. We note that the switch error rate from the ONT data and pipeline is of the order ~0.01 (see Shafin *et al*[7]), similar to what we observe here with SIGNALS, meaning we cannot determine accuracy beyond this level with this approach.

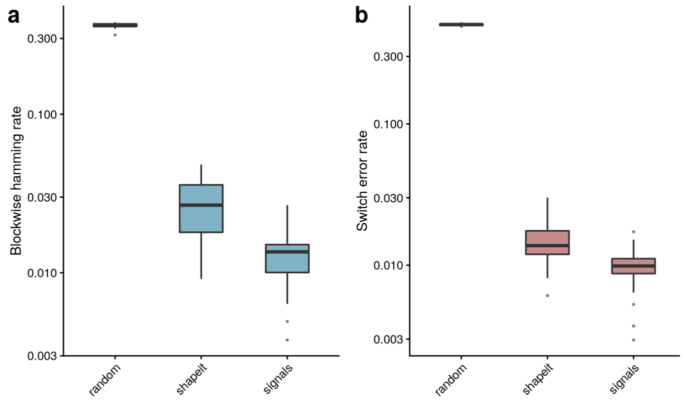

Supplementary Note Figure 7 Assessment of SNP phasing using blockwise hamming distance **a)** and switch error rate **b)** vs ONT used as ground truth. Each boxplot summarizes the distribution across the 22 autosomal chromosomes, n=22 per boxplot. All boxplots represent the median, 1st and 3rd quartiles (hinges), and the most extreme data points no farther than 1.5x the interquartile range from the hinge (whiskers).

Benchmarking against simulations

As a final benchmarking exercise we generated a simple simulation scheme to generate synthetic data where the ground truth is known. Our algorithm works as follows:

1. Generate haplotype-specific copy number profiles per chromosome arm.
   Input parameters:
   a. Which chromosomes are aberrant
   b. Allelic imbalance of each event
   c. Number of cells sharing the CNA
2. Convert the states to binned values across the genome
3. Sample the total number of haplotype counts per bin per cell
   a. Total number of counts follow a gamma distribution with shape 1.76 and rate 0.09 which we derived empirically from our data. This gives on average ~20 counts per bin
4. Given the haplotype-specific state, sample the B allele counts using a BetaBinomial model with total counts as input and p=BAF and user-specified overdispersion parameter, ρ. For LOH states BAF = BAF + ε, where ε is the LOH error rate
5. Randomly flip the counts of the B allele so that the counts are "unphased"
6. Output:
   a. Total copy number values in bins across the genome
   b. Unphased B allele counts
   c. Ground truth haplotype-specific copy number values in bins across the genome

We can then use outputs a) and b) as input to SIGNALS and assess our ability to recover the ground truth (output c)). We can also assess our ability to recover the inputted overdispersion and LOH error rate parameters.

We generated a number of simulations with varying levels of overdispersion (ρ), LOH error rates (ε) and varied the size of the smallest aneuploid clone. We were able to accurately infer both ε and ρ. Our accuracy in inferring ground truth haplotype-specific states was 99% with a modest decrease when aneuploid subclones were small, **Supplementary Note Fig.8**. We further discuss our ability to accurately infer haplotype-specific copy number in rare aneuploidies later in this document.

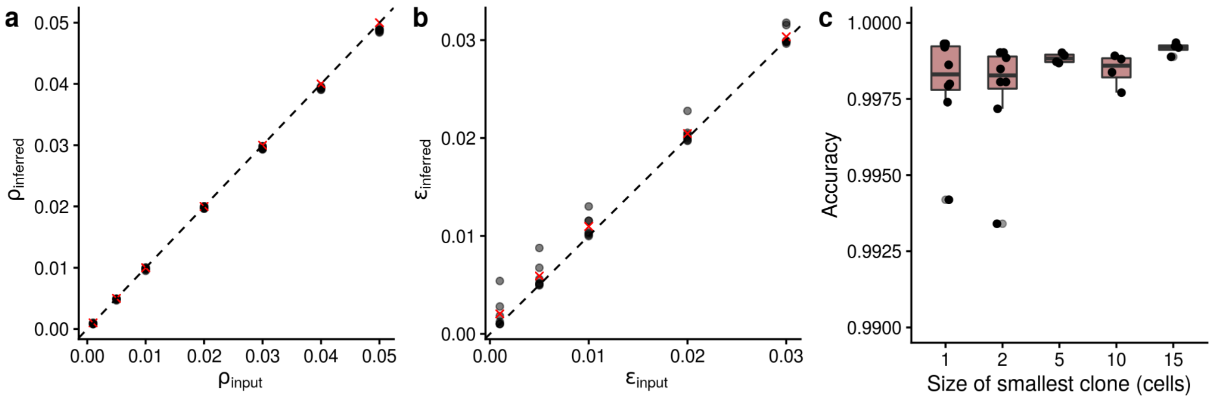

Supplementary Note Figure 8 Simulations
a) Inferred overdispersion parameter vs input overdispersion parameter. Each black dot is an individual simulation, red cross is the mean value from all simulations. Dashed black line is the y=x line. **b)** Inferred LOH error rate paremeter ε vs input values. **c)** Accuracy of inferred haplotype-specific copy number with varying clone sizes. Each dot is an individual simulation, (n=10

simulations per boxplot). All boxplots represent the median, 1st and 3rd quartiles (hinges), and the most extreme data points no farther than 1.5x the interquartile range from the hinge (whiskers).

Influence of changing the transition probability

We assessed the influence of the transition probability on our results using a range of values spanning from 0.1 to 0.999 and also including the case where there is no correlation structure between neighboring bins (IID). This was done using the OV2295 dataset (ovarian cancer cell line). We found that changing the transition probability has minimal effect on our results, even in the extreme case of comparing the results in the IID case versus p_T = 0.999 the similarity in assigned states is 95%. We found that when p_T > 0.5, the number of segments per cell and the distance between the raw BAF and expected BAF based on the assigned state converged to a stable value, **Supplementary Note Fig.9**. Given these results, we reason that any value > 0.5 gives acceptable results and use 0.95 as the default in SIGNALS. The ability to increase p_T can be useful in the case of noisy datasets. We used a value of 0.999 for SA1292 for this reason.

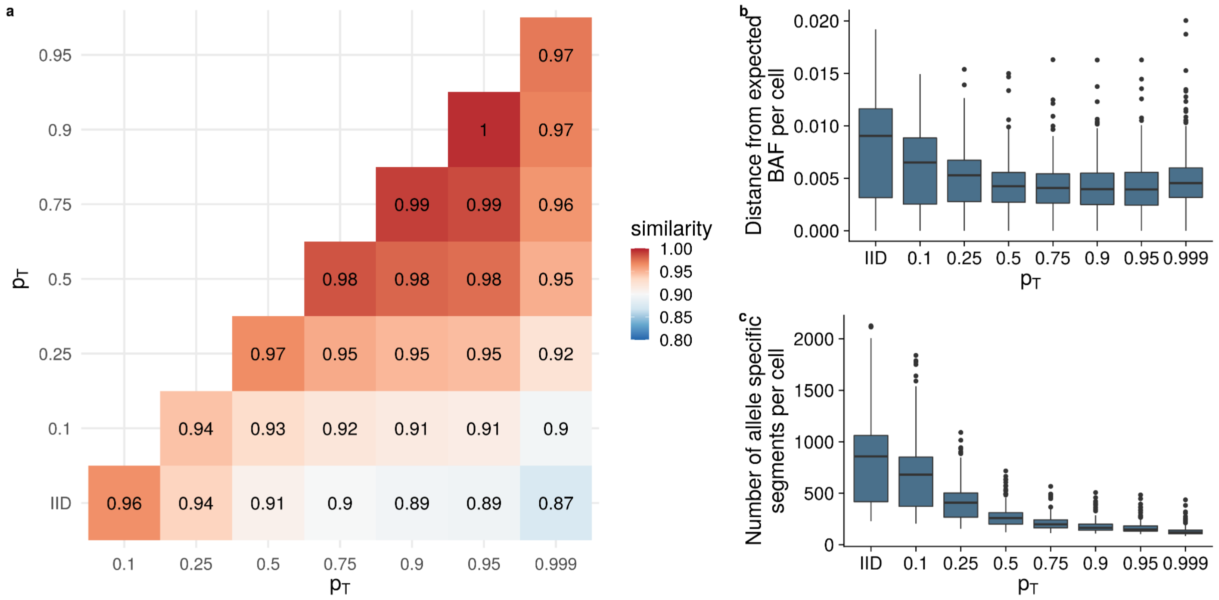

Supplementary Note Figure 9
a) Heatmap of similarity between haplotype-specific calls using different transition probabilities **b)** Distribution of the distance between the expected BAF and the empirical BAF as a function of transition probability. Each data point is the BAF value from a bin in a single cell (number of cells = 1084 cells, number of bins = 4325) **c)** Number of allele specific segment per cell as a function of the transition probability (number of data points per boxplot = number of cells = 1084). IID: independent and identically distributed, no correlation between neighbouring bins. All boxplots represent the median, 1st and 3rd quartiles (hinges), and the most extreme data points no farther than 1.5x the interquartile range from the hinge (whiskers).

Accurate haplotype phasing in events present in single cells

A key advantage of our approach is the ability to accurately phase and infer haplotype-specific copy number in rare cell populations. This is achieved by implementing a 2-step phasing approach where, in the second phasing step, only cells found to have allelic imbalance after the first step (or having odd number total copy number) are used for phasing. This allows us to use rare aneuploidies to accurately phase the

haplotype block alleles. To illustrate this, we will take a detailed look at the results from sample SA1047 which is a relatively homogeneous tumor, but has a number of rare aneuploidies.

A key parameter that can be modified in SIGNALS to adjust the sensitivity is the size of the smallest cluster that will be used for phasing. The default value is 10 but lowering this can be appropriate in some cases. We applied SIGNALS to SA1047 using the following cutoffs for the number of cells: 1,2,5,10 and also leaving the haplotype blocks unphased. From the total copy number, we can identify whole chromosome losses in a small number of cells on chromosomes 8 (7 cells), 13 (4 cells) and 11 (3 cells), **Supplementary Note Fig.10a**. Using 1 cell as the minimum cluster size, we could correctly phase these events into their respective homologs. Interestingly, in chromosome 13 these losses occurred on different homologs. BAF clearly skewed toward 0 or 1 in these cells when using 1 cell as our cluster size cutoff, **Supplementary Note Fig.10b,c**. In the case where we used 10 cells as the cutoff, there was some skew toward 0.0 or 1.0 but not as pronounced, while in the unphased case, values appeared to be distributed randomly between 0 and 1, **Supplementary Note Fig.10b,c**. Looking at the BAF in these rare aneupoidies on chromosomes 8, 11 and 13, they all were close to 0.0 and 1.0 with the 1 cell cutoff, confirming our ability to phase events and identify parallel copy number events present in very small numbers of cells.

We note that when using such smaller numbers for phasing, many haplotype blocks may be lost. This is because not every haplotype block is represented in every cell and therefore we will lose some by chance. In this example, we retained 92% of haplotype blocks, but in cases where the per cell coverage is low this may be much higher and can result in poor results. SIGNALS outputs this fraction of haplotypes retained as a diagnostic measure, so users should pay careful attention to this number when they lower the minimum cluster size.

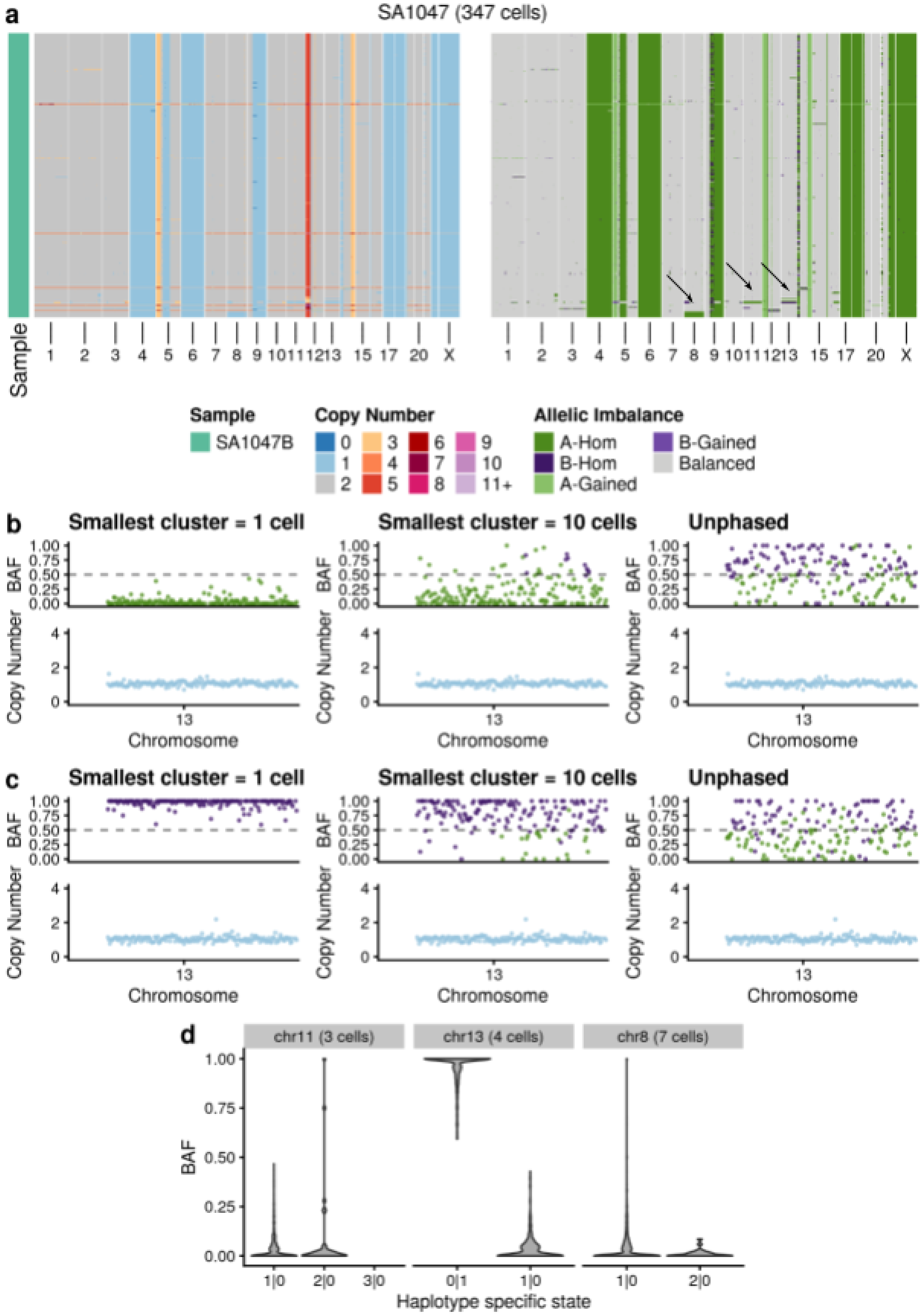

Supplementary Note Figure 10 Phasing rare populations in SA1047

a) Heatmap representation of sample SA1047. Left shows total copy number, right shows haplotype specific copy number states. Arrows show small aneuploid populations **b)** The same single-cell with a chromosome 13 loss with results shown when SIGNALS was run with a 1 cell cutoff, a 10 cell cutoff and unphased haplotypes. **c)** A different cell with a chromosome loss on 13, this time of the opposite haplotype. **d)** BAF distributions in the cells highlighted in panel a with chromosome losses

Comparison with other methods

Recently, two other methods have been published that also estimate haplotype specific copy number in sparse single cell whole genome sequencing. CHISEL[2] was the first method developed to specifically address this problem and is more similar to SIGNALS. It uses binned read depth ratios and BAF's computed from haplotype blocks and then jointly clusters these values to infer haplotype-specific copy number. Read depth ratios are computed using a matched normal. Differently to SIGNALS, haplotype blocks are defined over a user inputted range with 50Kb as the default. The assumption is that haplotype switches are rare at this length scale. The principle behind Alleloscope is to genotype segments in individual cells. A segmentation profile is generated using circular binary segmentation from the read depth ratio of a pseudobulk of all cells. Allelscope[9] uses SNPs rather than phased haplotype blocks, it first phases the SNPs using an EM algorithm and then for each segment in each cell identifies the most likely state based on the BAF and the RDR in the segment. See table 1 for a summary.

	**SIGNALS**	**CHISEL**	**Alleloscope**
**Algorithm**	HMM based on haplotype block read counts	Clustering + smoothing based on RDR and BAF	Phasing SNPs + genotyping segments
**Resolution**	0.5Mb	3-5Mb †	Pseudobulk derived segmentation
**Cell level segmentation**	✅	✅	❌
**Use of reference panel-based phasing**	✅ ‡	✅	❌
**State space**	Total copy number from 0-11	Unlimited	Total copy number from 0-6*
**Phasing of rare cell populations**	✅	❌	?
**Integration with scRNAseq**	✅	❌	❌
**Integration with scATACseq**	❌	❌	✅
**Computational requirements +**	Low	High	Low

Table 1 Comparison of algorithmic details and capabilities of the 3 tools

† This can be modified to be lower but for coverage of ~0.04X (this study) 3Mb is the lower limit recommended by the developers.
* Default is 0-6 but user can increase this. Visualization is capped at 6
‡ User can opt to use SNPs but this results in reduced performance
+ A quantitative benchmarking comparison of computation times is unfair due to differences in approaches. CHISEL has large computational requirements because it implements the read depth calculations and genotyping itself directly from the BAM files. This appears to be the most computational expensive step in terms of memory and time. These are input files for SIGNALS and Alleloscope. The time and memory requirements of the copy number calling are likely broadly similar across tools.

To compare the performance of SIGNALS relative to these other methods we ran both CHISEL and alleloscope on 12 of our datasets. We chose 12 datasets to encompass a range of tumor types and baseline ploidies. In addition, we also ran our pipeline on breast cancer patient S0, which was studied extensively in the CHISEL paper. We obtained the CHISEL results for this data from the original publication.

Running CHISEL and Alleloscope

We ran CHISEL with default parameters apart from using 10kb as haplotype block switching range (the -k parameter). The developers recommended this when using the 1000 genomes SNPs as the reference panel due to the quality of phasing being poorer with this approach relative to using the Haplotype Reference Consortium (HRC). We also ran CHISEL with a smaller bin size of 0.5Mb, in this case, we increased the ploidy sensitivity to 6 (-A parameter) as we found quite variable ploidy estimation at this resolution, though as shown below, CHISEL did not produce reliable estimates at this resolution, as expected. In the following analysis *chiselsmall* indicates the CHISEL run with smaller bin sizes. The input to CHISEL is a single bam file with barcoded single cells, to generate this we followed the steps outlined in the documentation. CHISEL also requires phased haplotypes, we used the SHAPEIT haplotypes generated in our pipeline, dropping the hap_label column that indicates uncertainty over phasing boundaries. We found that the memory and CPU requirements were very large for datasets with a large number of cells. Specifically, we tried to generate results for a dataset with >5000 cells but the program often ran out of memory with the maximum available on our HPC (500Gb) and failed to complete after 14 days running time. We, therefore, used a maximum of 1000 cells in each dataset.

Alleloscope was run with default parameters. The input to alleloscope are binned read counts across the genome in each cell and matched normal and heterozygous SNP counts in each cell. All of these are computed by CHISEL and outputted in the first steps of the CHISEL algorithm (rdr.tsv and baf.tsv) so we used these as input to Alleloscope.

For ease of comparison, we converted the output to be in the same format as SIGNALS. Copy number heatmaps from sample SA530 for all 4 methods are shown in **Supplementary Note Fig.11**. All these tables are available in the zenodo data repository for each dataset.

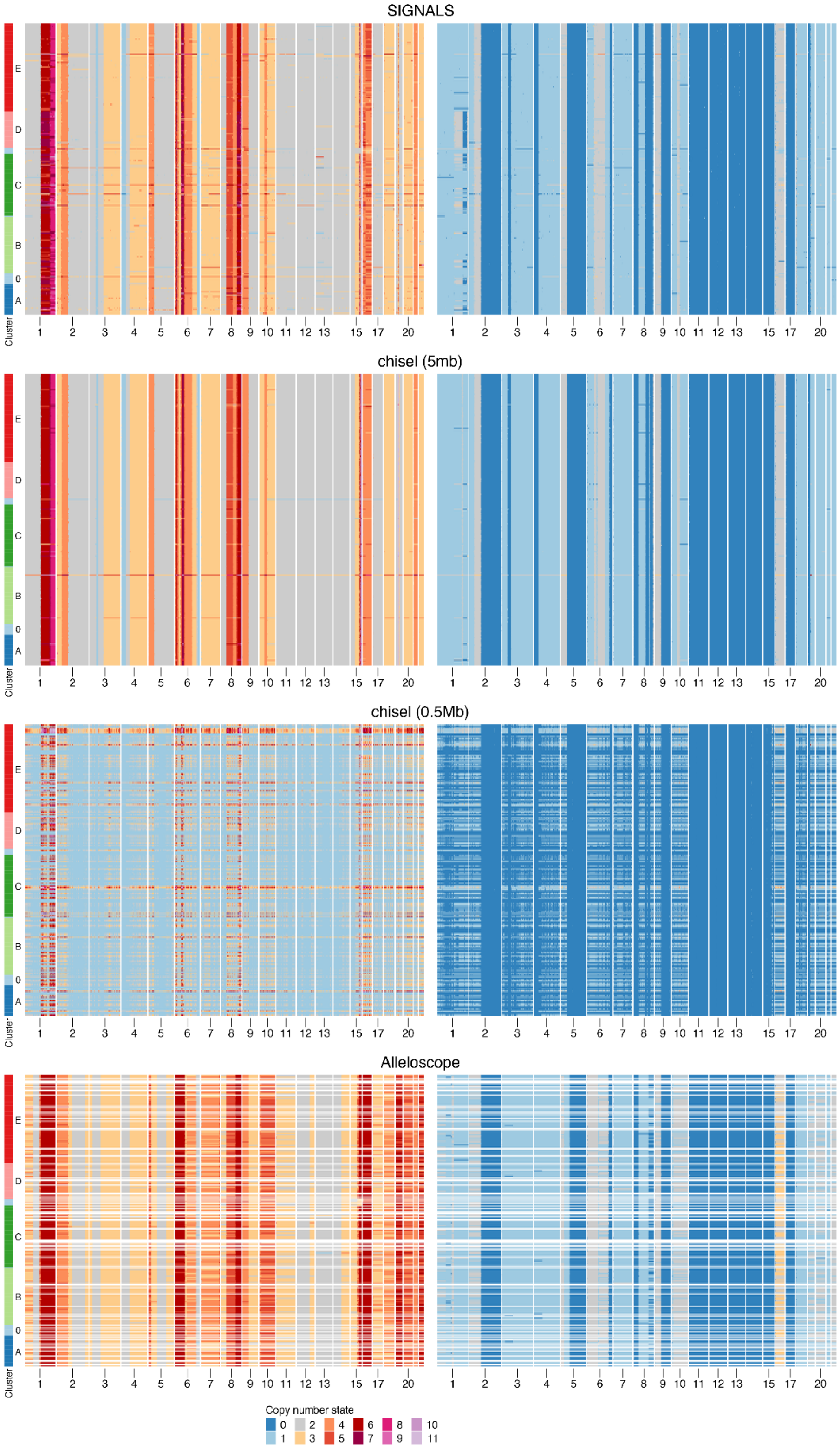

Supplementary Note Figure 11

Copy number heatmaps from 4 methods (SIGNALS, chisel, chisel with 0.5Mb bins - chiselsmall and alleloscope). Shown is total copy number, left and minor allele copy number, right. Colours according to legend at the bottom. White rows in heatmaps indicate that those cells were filtered out by the tool.

We first investigated how similar the reported allele-specific estimates were for each method. We compared the fraction of total copy number calls and minor allele copy number calls that were identical on a cell-by-cell and method-by-method basis. We found that SIGNALS and CHISEL with 5Mb were the most similar with an average minor allele copy number similarity of more than 90%. Alleloscope and chisel with 0.5Mb bin size were the least similar. **Supplementary Note Fig.12a**.

Next, we used a similar strategy to our overall validation of SIGNALS and compared allele-specific copy number derived from a matched bulk WGS sample with single-cell pseudobulk estimates. We found that there was a large discrepancy between estimated ploidy from alleloscope compared to CHISEL and SIGNALS which were most similar to the bulk derived ploidy estimates, **Supplementary Note Fig.12b**. We also observed that Alleloscope and CHISEL with 0.5Mb were less well correlated with the minor allele copy number, **Supplementary Note Fig.12c**.

As another metric of comparison, we computed variant allele frequency distributions per state and clustered these distributions to identify modes. We then calculated the distance from the expected mode to the closest identified mode. We used the CNAqc package for clustering and distance calculations. We found that CHISEL and SIGNALS generally had the smallest distance between expectation and observations. Finally, we looked at the proportion of SNV's in balanced and unbalanced states that had VAF > 0.95. Mutations with VAF > 0.95 would indicate a mutation residing in a region of loss of heterozygosity so a large fraction of mutations with VAF > 0.95 would indicate incorrect haplotype-specific copy number inference.

Together these results suggest that CHISEL with 5Mb bins and SIGNALS (0.5Mb) perform equally well at estimating allele-specific copy number. We did not observe a statistically observable difference in any of our metrics for these two methods. CHISEL at a lower bin size of 0.5Mb results in a loss of performance. Alleloscope appears to incorrectly infer baseline ploidy in many cases, incorrectly identifying whole-genome doubled tumors as diploid. This likely is the reason for the comparatively poorer performance. Alleloscope offers a function to re-assign states in a second stage estimation, which may improve performance in this scenario. This functionality (as far as we understood) requires defining a region as diploid heterozygous that could be used as a reference to rescale the copy number calls. However, in some of our samples we did not observe regions of the genome that were maintained in a heterozygous diploid state.

In summary, from our assessment of our benchmarking we conclude that SIGNALS is able to perform at least equally as well as CHISEL but at an order of magnitude greater genome resolution.

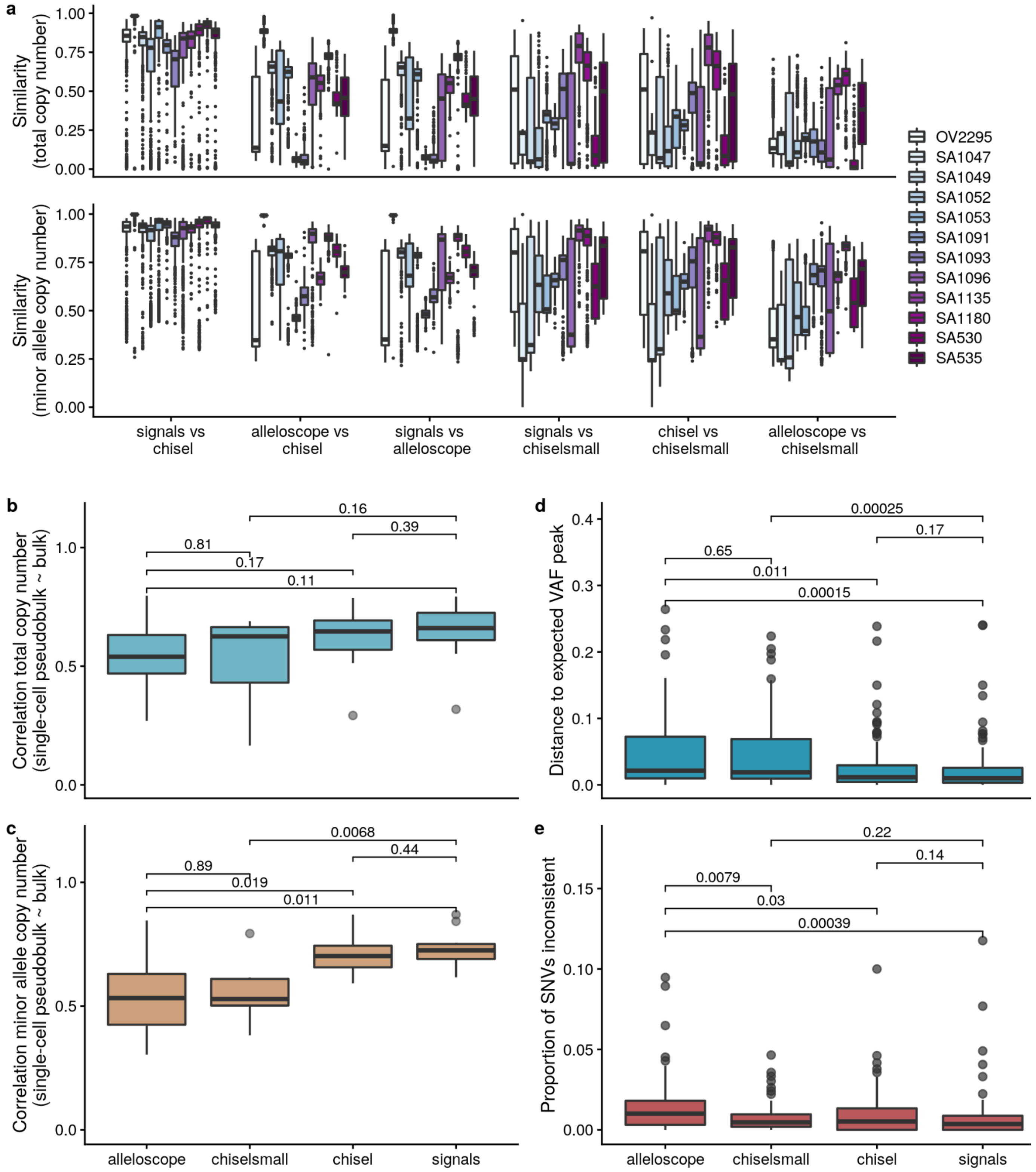

Supplementary Note Figure 12 Comparison of methods
a) Similarity of total and minor allele copy number calls between methods across 12 datasets. Each boxplot shows distribution of per cell similarity (n = 1000 cells) between methods. Comparisons are ordered by the average most similar (SIGNALS + CHISEL) to least similar (alleleoscope and CHISEL with 0.5Mb bins). **b)** Difference in bulk derived vs single cell pseudobulk derived ploidy estimates. Each data point is one of the 11 samples with a matched bulk WGS (not available for OV2295). **c)** correlation between the minor allele copy number in bulk vs single-cell derived pseudobulk **d)** Distance of expected VAF peak to

empirically observed VAF peaks. **e)** Proportion of SNVs in balanced or unbalanced states that have VAF > 0.95 indicative of missasigned states. In each boxplot panel, the numbers and labels indicate the p-values from pairwise Wilcoxon tests. **b)-e)** boxplots summaries values across 10 datasets. Pairwise p-values are derived from two sided Wilcoxon test. All boxplots represent the median, 1st and 3rd quartiles (hinges), and the most extreme data points no farther than 1.5x the interquartile range from the hinge (whiskers).

Comparing rare cell populations

One aspect that our analysis thus far does not directly address is the relative performance of the algorithms in calling CNAs which are only present in a small number of cells. Determining the accuracy of inferences in rare populations is challenging due to a lack of ground truth data. Assessing accuracy using bulk data will largely be informative of clonal events and using SNV VAF distribution is at best informative of subclones at high frequency. SNVs will mostly be too sparse if the cell number is low. We, therefore, turn to other measures and heuristics to determine the reliability of CNAs only identified in a small proportion of cells. One piece of information we can look at to at least check for consistency is the BAF of particular events in individual cells and the number phasing switches we observe in particular events. The rationale here is that if the phasing is inaccurate in small numbers of cells then this will result in nonsensical BAF and the dominant allele flipping between A and B across an event in a single cell. In our experience, accurate phasing is the key determinant of the ability to accurately resolve rare CNAs at haplotype resolution. To look at this in detail we compared the results of SIGNALS and CHISEL at 5Mb resolution in the data from patient S0 originally presented in CHISEL. We focussed on CHISEL as this showed the most comparable performance in our previous tests. We obtained CHISEL results for this sample from the original paper and obtained BAM files for this data from the 10X website and ran it through our HMMcopy + SIGNALS pipeline. These results, therefore, represent best use case scenarios as they were run by the developers of each tool.

Overall we found a high degree of consistency in calls, on average 93% of total copy number calls and minor allele copy number calls were identical in SIGNALS and CHISEL, **Supplementary Note Fig.13**. However, we do observe a small proportion of cells that are considerably divergent. Many of these cells are due to different baseline ploidy assignments between the two methods.

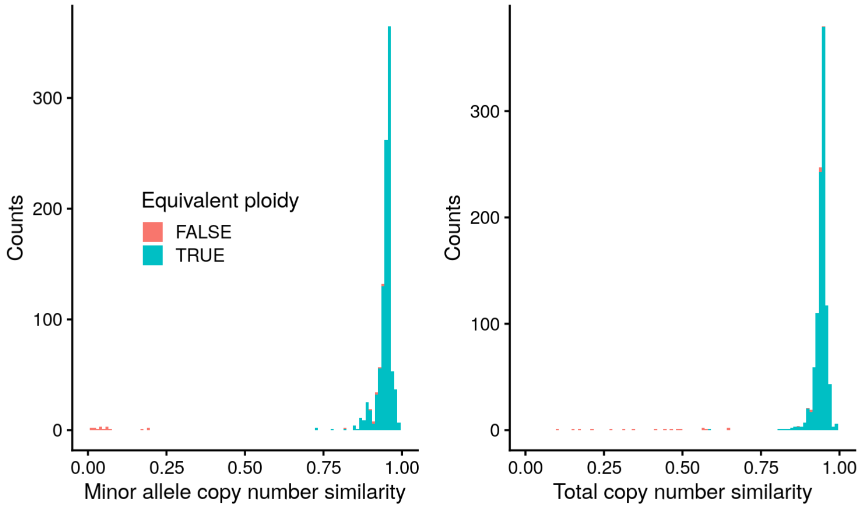

Supplementary Note Figure 13
Histograms of the similarity between minor allele copy number and total copy number between CHISEL and SIGNALS.

Over and above these cells where ploidy assignment differed we also observed CNA calls in CHISEL where the BAF appeared to be inconsistent with the assigned state and switching between the dominant allele, **Supplementary Note Fig.14**. In our experience, this is indicative of incorrect phasing.

In summary, this investigation of patient S0 suggests that SIGNALS is able to better phase haplotypes when CNAs are rare resulting in improved haplotype-specific copy number in rare populations and identification of rare mirrored imbalances.

A note on GC bias and correction

Neither CHISEL nor Alleloscope perform GC correction of read counts. They rely on normalization of read counts by taking read depth ratios between tumor cells and either a matched bulk normal or pseudobulk of normal cells derived from the same sample of sequenced cells. This relies on the GC bias being similar between tumor cells and the corresponding normal. This is a reasonable assumption when the library preparation methods are identical, for example when using normal diploid cells as controls sequenced within the same experiment. However, in much of our data, our tumor cells are prepared using our bespoke DLP assay and we use a bulk normal prepared using a standard whole genome sequencing library preparation protocol. This mismatch may mean a simple ratio-based normalization may not adequately remove GC bias. Our investigations showed that in certain samples this was particularly problematic at smaller bin sizes, **Supplementary Note Fig.15**. This may explain in part the poor performance of CHISEL when used with a smaller bin size. Correcting read counts due to GC bias prior to input to the CHISEL algorithm may produce improved performance when using smaller bin sizes. We also note that other sources of biological variability such as the replication state of cells may introduce GC bias, we, therefore, think that GC correction is strongly advisable for single cell whole genome analysis.

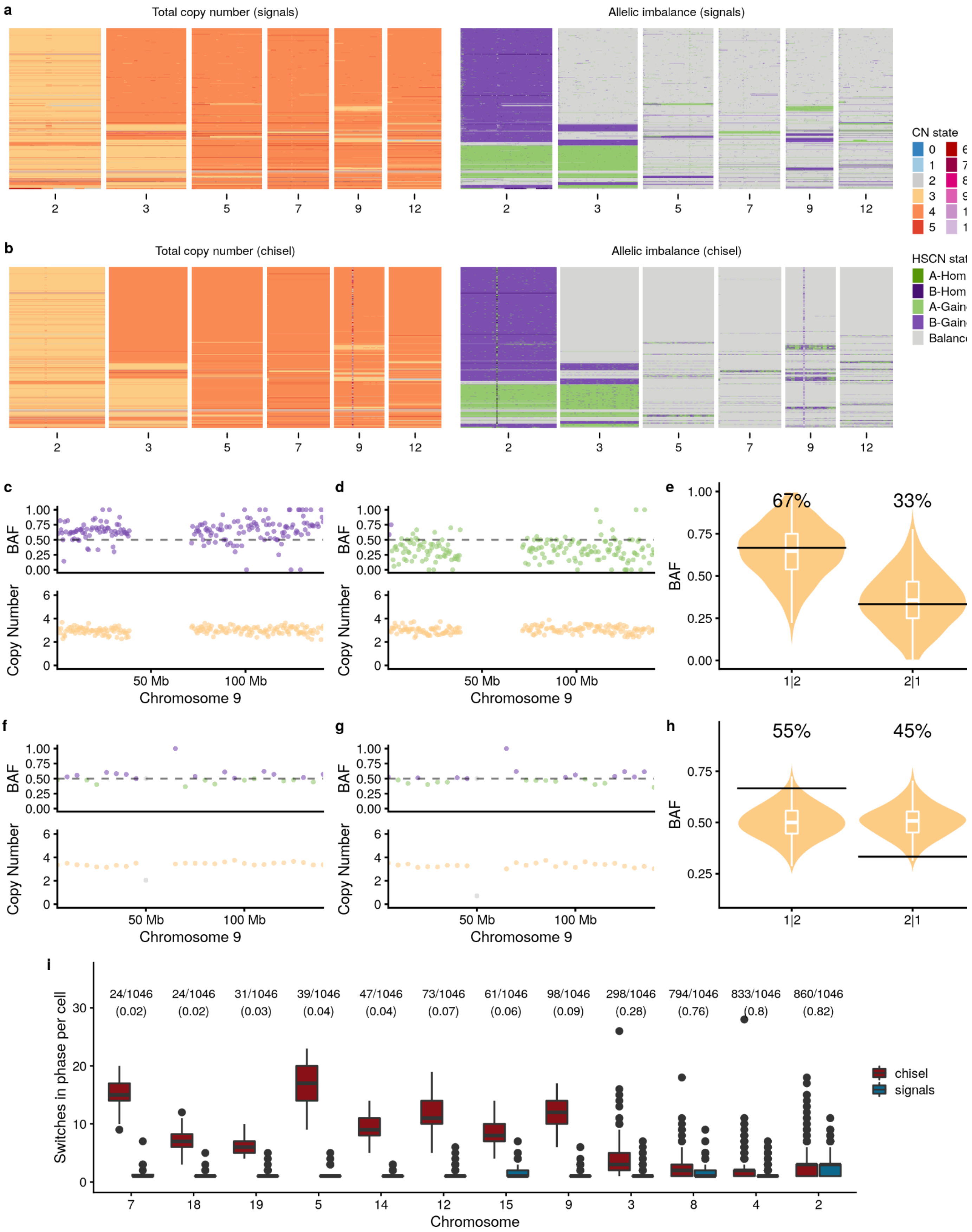

a
Total copy number (signals)
Allelic imbalance (signals)
2 3 5 7 9 12
CN state
0 1 2 3 4 5
HSCN stat
A-Hom
B-Hom
A-Gain
B-Gain
Balanc
b
Total copy number (chisel)
Allelic imbalance (chisel)
c
d
e
BAF
Copy Number
Chromosome 9
50 Mb 100 Mb
67%
33%
1|2
2|1
f
g
h
55%
45%
i
24/1046 (0.02)
24/1046 (0.02)
31/1046 (0.03)
39/1046 (0.04)
47/1046 (0.04)
73/1046 (0.07)
61/1046 (0.06)
98/1046 (0.09)
298/1046 (0.28)
794/1046 (0.76)
833/1046 (0.8)
860/1046 (0.82)
Switches in phase per cell
7 18 19 5 14 12 15 9 3 8 4 2
Chromosome
chisel
signals

Supplementary Note Figure 14
Heatmap representation of all cells from section E patient S0 for a subset of chromosomes from SIGNALS **a)** and CHISEL **b)**. Left hand heatmap is totla copy number, right is the haplotype specific copy number state. Note that in **b)** chr5,7,9&12 the triploid chromosomes flip between green and purple while in **a)** colours are continuous across a chromosome in an individual cell. **c,d** Two single cells with mirrored allelic imbalance in chromosome 9 inferred with SIGNALS. **e)** BAF distributions of cells with allelic imbalance in chromosome 9 from SIGNALS. Black horizontal line indicate the expected means of the distributions. **f,g** The same 2 single cells inferred with CHISEL showing inconsistent BAF given triploid state and switching of phase across the chromosome. Number of data points in **e**: 11764 bins in 1|2 state and 5274 bins in 2|1 state. Number of data points in **f**: 1457 bins in 1|2 state and 1064 bins in 2|1 state. **h)** BAF distributions of cells with allelic imbalance in chromosome 9 from CHISEL **i)** The number of switches in phase in individual cells across chromosomes. Each data represent the number of switches in a chromosome in an individual cell. The number across the top indicates the number and fraction of cells that are aneuploid in that chromosome according to SIGNALS, boxplot shows the distribution of switches in those aneuploid cells. All boxplots represent the median, 1st and 3rd quartiles (hinges), and the most extreme data points no farther than 1.5x the interquartile range from the hinge (whiskers).

SIGNALS with scRNAseq

SIGNALS also facilitates integration with scRNAseq data in order to assess allelic imbalance in the transcriptome. We called heterozygous SNPs in the scRNAseq data using cellSNP. As input, we used the same set of heterozygous SNPs identified in the scDNAseq and the corresponding normal sample for each sample. The liftover script provided in cellSNP was used to lift over SNPs from hg19 to hg38. Following genotyping, we phase the SNPs using the phasing information computed from the allele specific inference in the scDNAseq. SNP counts are much more sparse in scRNAseq vs scDNAseq (~2 orders of magnitude lower), estimates of allelic imbalance at a scale of 0.5Mb is therefore not possible in scRNAseq data. As an alternative, we computed a consensus copy number segmentation, using the functions `create_segments` and `consensuscopynumber` in SIGNALS. Segments smaller than 10Mb were smoothed using the `filter_segments` function. Phased SNP counts were then aggregated per segment per cell to estimate allelic imbalance in each segment. Applying this to over 85,000 single cells from 14 datasets, we found that the mean and the variance of BAF per segment were highly correlated across all the samples, **Supplementary Note Fig.16**.

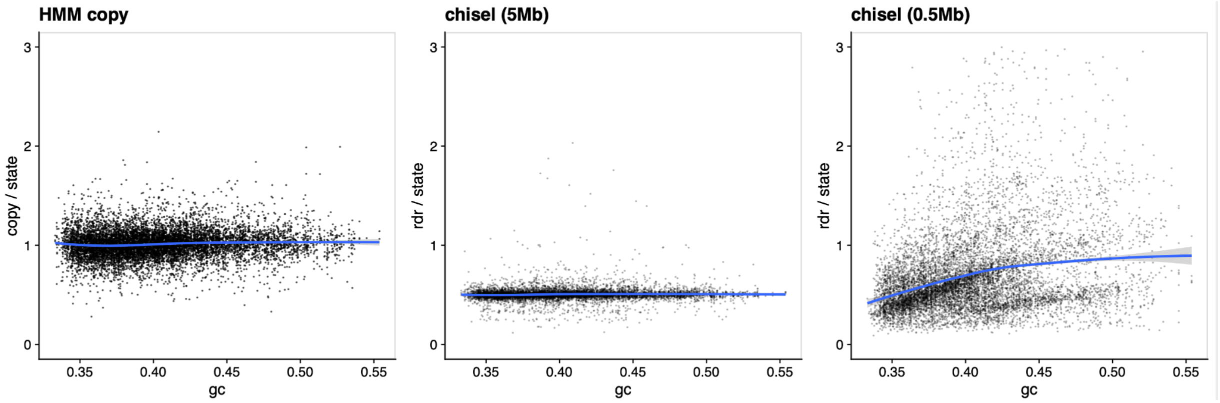

Supplementary Note Figure 15 GC bias
Copy values (GC bias corrected read counts) divided by the inferred total copy number state as a function of GC content from HMMcopy output for sample SA530. b rdr divided by inferred total copy number state from CHISEL output at 5Mb bins c rdr divided by inferred total copy number state from CHISEL output at 0.5Mb bins. Lines in each plot show a GAM regression, with shaded areas indicating the 95% confidence interval.

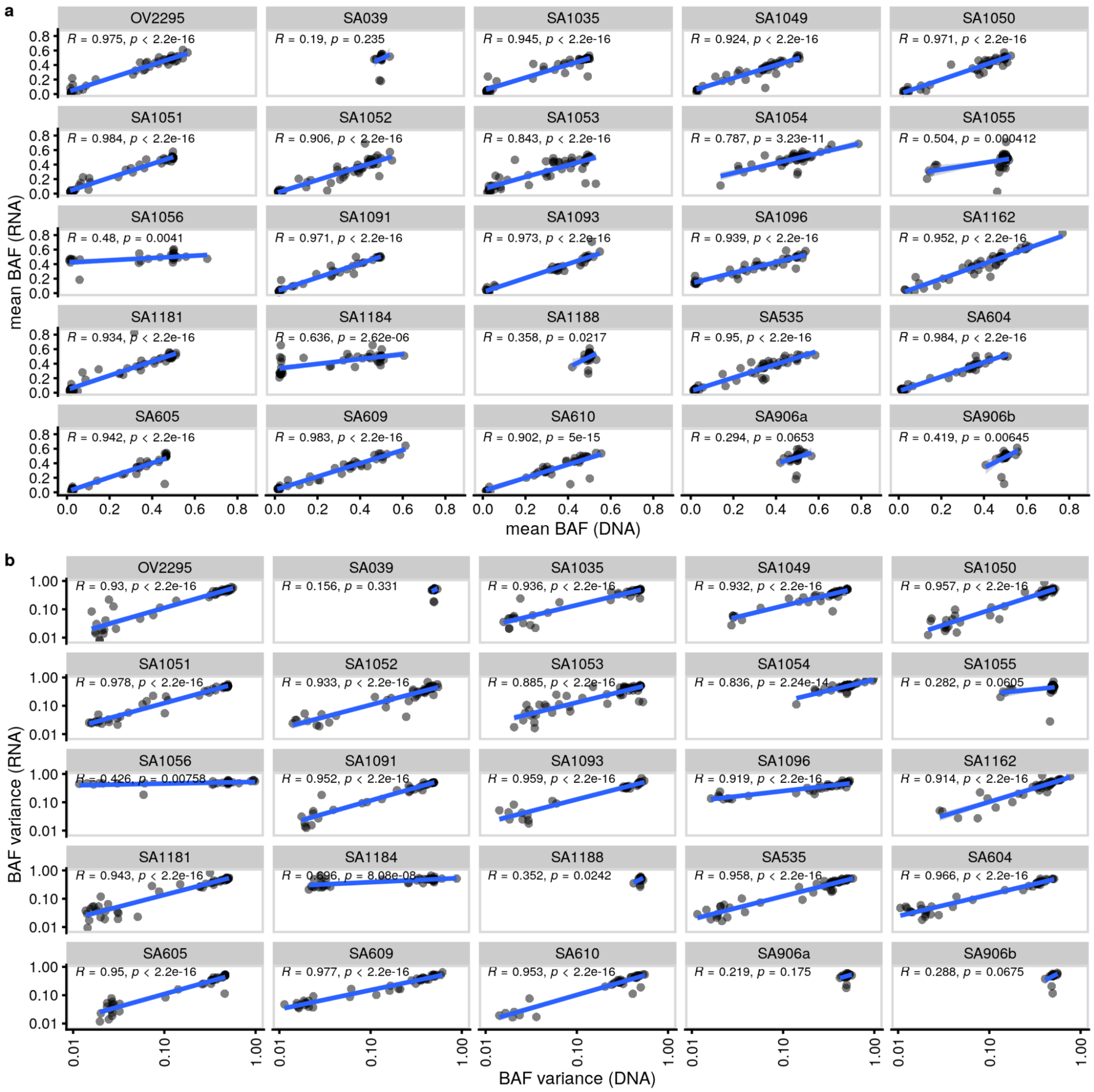

Supplementary Note Figure 16 a) Mean BAF per segments in scRNA (y-axis) vs scDNA (x-axis) **b)** Variance of BAF per segment in scRNA (y-axis) vs scDNA (x-axis). Annotations indicate the correlation coefficient (R) and p-value (p) derived from a linear regression using the lm function in R

Discussion of tools

Conceptually, SIGNALS and CHISEL are the most similar in terms of approach, and it is therefore perhaps unsurprising that we observed the highest degree of similarity between these methods. Both methods used a binning approach followed by some kind of smoothing, a HMM in the case of SIGNALS and clustering followed by smoothing in the case of CHISEL. Alleloscope requires the identification of heterozygous diploid regions that are used as a control for correct ploidy adjustment. Many of our tumors had highly complex high ploidy karyotypes where often a limited amount of the genome remained heterozygous diploid (confirmed by bulk WGS as shown above). In addition, as we primarily used cell lines or PDX models, typically no normal cells were sequenced which could be used for this purpose. In addition, one of the aspects unique to Alleloscope is that it uses a pre-determined segmentation and then frames the problem as genotyping all segments in all cells. This will result in underestimating heterogeneity in highly heterogeneous complex tumours, where the assumption that all cells share a similar segmentation profile is invalid. In summary, many of our samples included highly complex, high ploidy samples with differences in segmentation between clones and cells and a lack of diploid control cells. These aspects of our data may explain the relative decrease in performance for Alleloscope presented in this study. Alleloscope is, therefore, more appropriate for more homogeneous datasets where the tool can better take advantage of pooling across large genomic regions without loss of performance due to subclonal differences in segments and lack of diploid control regions.

In addition, Alleloscope works with scATACseq, while SIGNALS works with scRNAseq. In principle, the same approach used for scRNAseq in SIGNALS could be extended to scATACseq. We also note the results using SIGNALS benefits from careful data curation and filtering first described in Laks *et al*. This includes identifying cells undergoing DNA replication and filtering them out from downstream analysis, GC correction of read counts and removing problematic bins such as those with low mappability. In our experience, all of these aspects can have a considerable effect on downstream analysis and we would contend that most analyses of scDNAseq datasets would benefit from consideration of these issues.

Limitations of SIGNALS

While we show here that SIGNALS represents an advance in terms of genomic and cellular resolution of events in single cells there are still some limitations to be aware of. One challenge, in particular, is when chromosomes are highly complex with a large number of overlapping events, and there are no cells/clones with simple whole chromosome events that provide unambiguous phasing information. In these cases, phasing events to individual homologs may be undetermined and we recognize that we may have assigned events to the incorrect homologs in some cases. An elegant way to approach this, first suggested by Schwarts et al[10] and also utilized by CHISEL is to phase events in such a way that the number of haplotype specific segments with different copy number is minimized. CHISEL implements this via an efficient dynamic programming algorithm, we include an implementation of this in SIGNALs which can be accessed using the `rephasebins` function.

Additional Methods

Breakpoint calling in single cells

In order to assess our ability to recover SV's in pseudobulk clones we calculated the sensitivity of our approach using data from an ovarian cancer cell line. This data consists of cell lines generated from 3 sites at different time points (referred to as OV2295 in the text), meaning we can confidently identify breakpoints present in 100% of cells (those that are found in all 3 samples). We sampled groups of cells of varying size, calculated the cumulative coverage of these groups and then assessed the fraction of breakpoints we recover. We classified a breakpoint as "recovered" if any cell within the group of sampled cells had evidence of the breakpoint. **Supplementary Note Fig.17** shows the sensitivity as a function of cumulative coverage. At a cumulative coverage of ~5X we recover 80% of the breakpoints that are present, ~5X coverage translates to approximately 100-150 cells given our median coverage of 0.04X per cell.

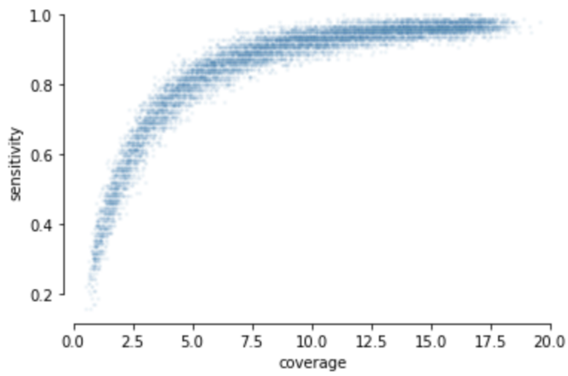

Supplementary Note Figure 17
Sensitivity of structural variant recall as a function of the cumulative coverage of randomly sampled cells that are merged to form a pseudobulk.

Oxford nanopore long read sequencing

To ensure consistency between DLP scWGS and nanopore we compared consensus copy number profiles dervied from DLP with copy number estimates dervied from ONT. For ONT, copy number was estimated using the QDNASeq package[11]. This showed high similarity between datasets, see **Supplementary Note Fig.18** for the SA1096 dataset. We also validated that we could recover clonal breakpoints (present in 100% of cells) predicted from DLP pseudobulk in ONT data. **Supplementary Note Fig.19** shows an example of a clonal interchromosomal rearrangement in DLP pseudobulk and ONT long read data.

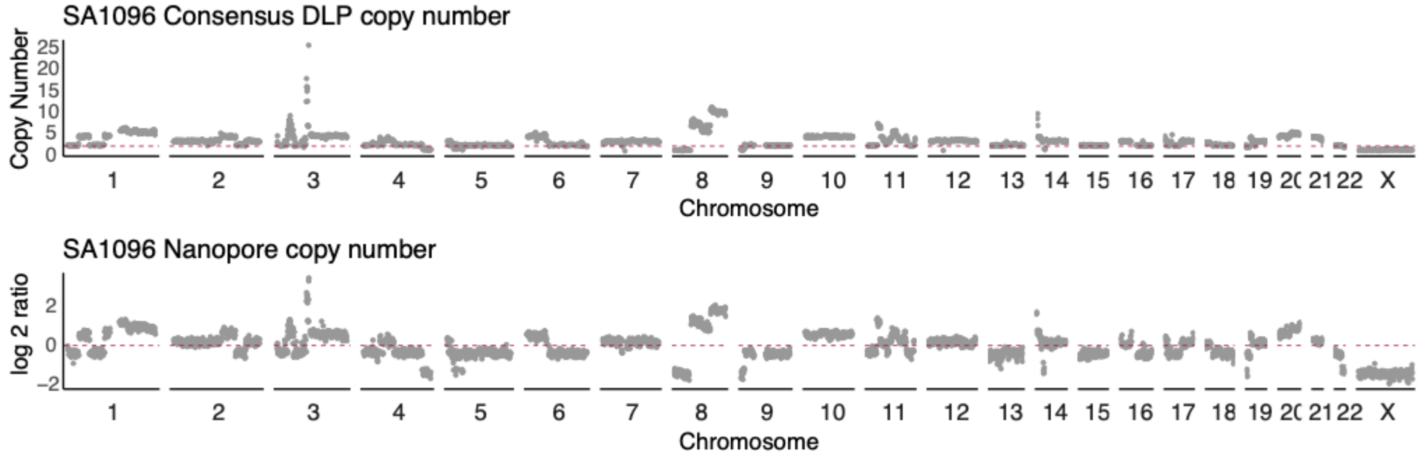

Supplementary Note Figure 18
Copy number profiles from DLP pseudobulk and ONT long read data across the whole genome for SA1096. Each data point is the normalized copy number or log2 ratio in 0.5Mb bins across the genome.

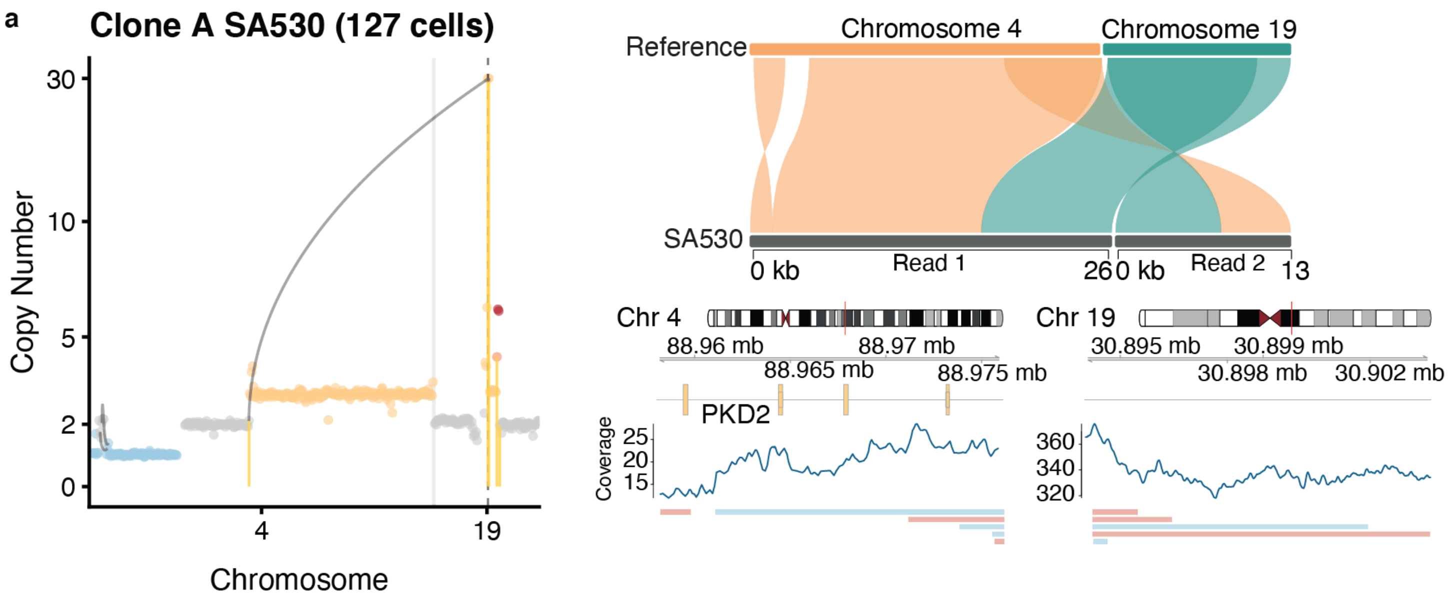

Supplementary Note Figure 19
Interchromosomal rearrangement adjacent to a clonal high level amplification in DLP and ONT. Left plot shows copy number profiles from a pseudobulk clone derived from DLP, lines indicate rearrangement breakpoints, right plot shows example long reads from ONT that support the inter-chromosomal translocations. Example reads and their mapping to chromosomes of interest topright, long read coverage of genomic region and alignment of all supporting reads (bottom right).

Allele bias in event rate calculation

We used ancestral state reconstruction to estimate the event rate of gains and losses (see main text and methods). To evaluate whether there was a particular bias for either haplotype we augmented this analysis by calculating the event rate of gains and losses per haplotype and then taking the ratio (Haplotype bias), expecting an average value of 1 if there was no bias toward either haplotype. Because SIGNALS has a bias toward assigning the minor allele as the A haplotype we randomly flipped the A and B haplotypes before performing this analysis. This revealed that on average there was no apparent bias toward losing or gaining a particular haplotype **Supplementary Note Fig.20**, although we cannot rule out that this happens in a minority of cases and is obscured in this averaged analysis.

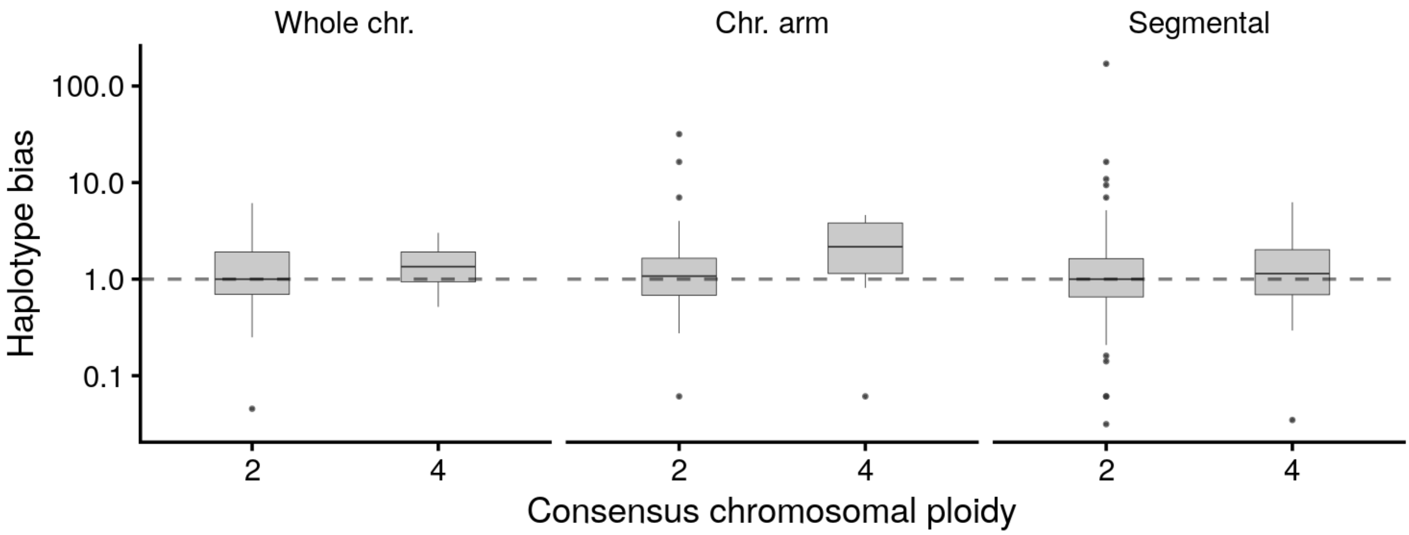

Supplementary Note Figure 20

Haplotype bias across events. Same events presented in main figure 5j. Number of events: whole chromosomes (n=35 events), chromosome-arms (n=31 events) and segments (n=341 events). All boxplots represent the median, 1st and 3rd quartiles (hinges), and the most extreme data points no farther than 1.5x the interquartile range from the hinge (whiskers).

References

1. Laks, E. *et al.* Clonal Decomposition and DNA Replication States Defined by Scaled Single-Cell Genome Sequencing. *Cell* **179**, 1207–1221.e22 (2019).
2. Zaccaria, S. & Raphael, B. J. Characterizing allele- and haplotype-specific copy numbers in single cells with CHISEL. *Nat. Biotechnol.* **39**, 207–214 (2021).
3. McPherson, A. W. *et al.* ReMixT: clone-specific genomic structure estimation in cancer. *Genome Biol.* **18**, 140 (2017).
4. Delaneau, O., Marchini, J. & Zagury, J.-F. A linear complexity phasing method for thousands of genomes. *Nat. Methods* **9**, 179–181 (2011).
5. Yee, T. W. The VGAM package. *R News* **8**, 28–39 (2008).
6. Tarone, R. E. Testing the goodness of fit of the binomial distribution. *Biometrika* **66**, 585–590 (1979).
7. Shafin, K. *et al.* Haplotype-aware variant calling with PEPPER-Margin-DeepVariant enables high accuracy in nanopore long-reads. *Nat. Methods* (2021) doi:10.1038/s41592-021-01299-w.
8. Martin, M. *et al.* WhatsHap: fast and accurate read-based phasing. doi:10.1101/085050.
9. Wu, C.-Y. *et al.* Integrative single-cell analysis of allele-specific copy number alterations and chromatin accessibility in cancer. *Nat. Biotechnol.* (2021) doi:10.1038/s41587-021-00911-w.
10. Schwarz, R. F. *et al.* Phylogenetic quantification of intra-tumour heterogeneity. *PLoS Comput. Biol.* **10**, e1003535 (2014).
11. Scheinin, I. *et al.* DNA copy number analysis of fresh and formalin-fixed specimens by shallow whole-genome sequencing with identification and exclusion of problematic regions in the genome assembly. *Genome Res.* **24**, 2022–2032 (2014).
