# Supplementary material for: Single-cell genomic variation induced by mutational processes in cancer
Source: Nature. 2022 Oct 26;612(7938):106–15. doi: 10.1038/s41586-022-05249-0 (PMC9712114; doi:10.1038/s41586-022-05249-0)
Supplement: Supplementary file 2 — Reporting Summary [file 41586_2022_5249_MOESM2_ESM.pdf]

nature portfolio

Corresponding author(s): Sohrab P Shah, Samuel Aparicio, Marc J Williams

Last updated by author(s): Sep 16, 2022

# Reporting Summary

Nature Portfolio wishes to improve the reproducibility of the work that we publish. This form provides structure for consistency and transparency in reporting. For further information on Nature Portfolio policies, see our Editorial Policies and the Editorial Policy Checklist.

## Statistics

For all statistical analyses, confirm that the following items are present in the figure legend, table legend, main text, or Methods section.

| n/a | Confirmed | |
|---|---|---|
| ☐ | ☒ | The exact sample size (*n*) for each experimental group/condition, given as a discrete number and unit of measurement |
| ☐ | ☒ | A statement on whether measurements were taken from distinct samples or whether the same sample was measured repeatedly |
| ☐ | ☒ | The statistical test(s) used AND whether they are one- or two-sided<br>*Only common tests should be described solely by name; describe more complex techniques in the Methods section.* |
| ☐ | ☒ | A description of all covariates tested |
| ☐ | ☒ | A description of any assumptions or corrections, such as tests of normality and adjustment for multiple comparisons |
| ☐ | ☒ | A full description of the statistical parameters including central tendency (e.g. means) or other basic estimates (e.g. regression coefficient) AND variation (e.g. standard deviation) or associated estimates of uncertainty (e.g. confidence intervals) |
| ☐ | ☒ | For null hypothesis testing, the test statistic (e.g. *F*, *t*, *r*) with confidence intervals, effect sizes, degrees of freedom and *P* value noted<br>*Give P values as exact values whenever suitable.* |
| ☐ | ☒ | For Bayesian analysis, information on the choice of priors and Markov chain Monte Carlo settings |
| ☒ | ☐ | For hierarchical and complex designs, identification of the appropriate level for tests and full reporting of outcomes |
| ☒ | ☐ | Estimates of effect sizes (e.g. Cohen's *d*, Pearson's *r*), indicating how they were calculated |

*Our web collection on statistics for biologists contains articles on many of the points above.*

## Software and code

Policy information about availability of computer code

| | |
|---|---|
| Data collection | DLP+ single cell WGS pipeline: https://github.com/shahcompbio/single_cell_pipeline<br>Bulk WGS pipeline: https://github.com/shahcompbio/wgs |
| Data analysis | MMCTM method: https://github.com/shahcompbio/MultiModalMuSig.jl v0.3.0<br>SIGNALS: https://github.com/shahcompbio/signals v0.7.2<br>CELLRANGER https://support.10xgenomics.com/single-cell-gene-expression/software v3.1.0<br>cellSNP https://github.com/single-cell-genetics/cellsnp-lite v1.2.2<br>seurat https://satijalab.org/seurat/ v4.1.0<br>scrublet https://github.com/swolock/scrublet v0.2.3<br>CHISEL https://github.com/raphael-group/chisel v1.0.0<br>Alleloscope https://github.com/seasoncloud/Alleloscope<br>sitka https://github.com/UBC-Stat-ML/sitkatree<br>castor https://cran.r-project.org/web/packages/castor/index.html v1.6.6<br>Guppy https://nanoporetech.com/nanopore-sequencing-data-analysis v3<br>minmap2 https://github.com/lh3/minimap2 v2.24<br>sniffles https://github.com/fritzsedlazeck/Sniffles v1.0.12<br>cuteSV https://github.com/tjiangHIT/cuteSV v1.0.11 |

For manuscripts utilizing custom algorithms or software that are central to the research but not yet described in published literature, software must be made available to editors and reviewers. We strongly encourage code deposition in a community repository (e.g. GitHub). See the Nature Portfolio guidelines for submitting code & software for further information.

# Data

Policy information about availability of data

All manuscripts must include a data availability statement. This statement should provide the following information, where applicable:

- Accession codes, unique identifiers, or web links for publicly available datasets
- A description of any restrictions on data availability
- For clinical datasets or third party data, please ensure that the statement adheres to our policy

All data are available for general research use. Processed data including somatic mutation data for bulk WGS, total (and allele-specific) copy number profiles for DLP + data and filtered count matrices for scRNA-seq data are available for download at https://zenodo.org/record/6998936 . Raw scRNA-seq data are available for download at https://ega-archive.org/studies/EGAS00001006343 . Raw single-cell sequencing data generated for this study are available from https://ega-archive.org/studies/EGAS00001006343 , and previously published single-cell sequencing data used in this study are available at https://ega-archive.org/studies/EGAS00001004448 and https://ega-archive.org/studies/EGAS00001003190 . Somatic mutation calls from bulk WGS for 16 patients with TNBC for whom the IRB consent does not include public deposition of raw sequencing data are available at http://www.ncbi.nlm.nih.gov/projects/gap/cgi-bin/study.cgi?study_id=phs003038.v1.p1, and raw sequencing data can be provided upon request under material transfer agreement to shahs3@mskcc.org. Bulk WGS BAM files from patients under IRB consent protocols for public release of raw data are available for download at https://ega-archive.org/studies/EGAS00001006343 , http://www.ncbi.nlm.nih.gov/projects/gap/cgi-bin/study.cgi?study_id=phs003036.v1.p1 and https://ega-archive.org/datasets/EGAD00001003268 (for previously published data) or by request under material transfer agreement to shahs3@mskcc.org and saparicio@bccrc.ca.

# Field-specific reporting

Please select the one below that is the best fit for your research. If you are not sure, read the appropriate sections before making your selection.

☒ Life sciences ☐ Behavioural & social sciences ☐ Ecological, evolutionary & environmental sciences

For a reference copy of the document with all sections, see nature.com/documents/nr-reporting-summary-flat.pdf

# Life sciences study design

All studies must disclose on these points even when the disclosure is negative.

| | |
|---|---|
| Sample size | 309 patients, 309 with bulk WGS and 23 with scWGSor RNAseq. 8 hTERT lines, 2 ovarian cancer cell lines. Sample size was determined by the number of patient tissues accrued and number of 184hTERT clones with confirmed knockout identified. |
| Data exclusions | poor quality and s-phase cells were removed from analysis as per Methods |
| Replication | Where possible, biological duplicates were sequenced by scDNAseq. 11 patients with scWGS have more than 1 replicate tissue sequenced by DLP+ |
| Randomization | Patients were stratified into groups according to their mutational signature as described in the manuscript. No grouping of animals was performed, so no randomization was necessary |
| Blinding | Patient tissues were stratified by mutational signature during analysis, blinding was not necessary during data collection or analysis as no groups were assigned. |

# Reporting for specific materials, systems and methods

We require information from authors about some types of materials, experimental systems and methods used in many studies. Here, indicate whether each material, system or method listed is relevant to your study. If you are not sure if a list item applies to your research, read the appropriate section before selecting a response.

## Materials & experimental systems

| n/a | Involved in the study | |
|---|---|---|
| ☐ | ☒ | Antibodies |
| ☐ | ☒ | Eukaryotic cell lines |
| ☒ | ☐ | Palaeontology and archaeology |
| ☐ | ☒ | Animals and other organisms |
| ☐ | ☒ | Human research participants |
| ☒ | ☐ | Clinical data |
| ☒ | ☐ | Dual use research of concern |

## Methods

| n/a | Involved in the study | |
|---|---|---|
| ☒ | ☐ | ChIP-seq |
| ☒ | ☐ | Flow cytometry |
| ☒ | ☐ | MRI-based neuroimaging |

# Antibodies

| | |
|---|---|
| Antibodies used | mouse anti-p53 (Santa Cruz SC-126), mouse anti-BRCA1 (Santa Cruz SC-6954), mouse anti-BRCA2 (Millipore OP95), goat anti-GAPDH (SC-48166), rabbit KRAS (Lifespan Bioscience, LS-B4683) |
| Validation | KRAS - Validated by our lab using human uterine tissue and by Lifespan Bioscience using human uterine and placental tissues. GAPDH - Validated by Santa Cruz. p53 - Validated by Santa Cruz using A549, Daudi, NTERA2, SW480 cell lines and human bladder carcinoma tissue. BRCA1 - Validated by Santa Cruz using A-431, HeLa, U2OS and MCF7 cell lines. BRCA2 - Validated by MIllipore using MCF7 cell line. |

# Eukaryotic cell lines

Policy information about cell lines

| | |
|---|---|
| Cell line source(s) | 184hTERT L9 (SA039), 184-hTERTTP53-/- (SA906a and SA906b), 184-hTERTTP53-/-;BRCA1+/- (SA1292), 184-hTERTTP53-/-;BRCA1-/- (SA1054), 184-hTERTTP53-/-;BRCA2-/- (SA1055 and SA1056) and 184-hTERTTP53-/-;BRCA2+/- (SA1188), OV2295, TOV2295 |
| Authentication | All cell lines were authenticated by short tandem repeat testing by Genetica Lab Corp DNA Identity |
| Mycoplasma contamination | All cells were tested for mycoplasma contamination by Genetica Lab Corp DNA Identity and tested negative |
| Commonly misidentified lines (See ICLAC register) | No commonly misidentified cell lines were used in this study. |

# Animals and other organisms

Policy information about studies involving animals; ARRIVE guidelines recommended for reporting animal research

| | |
|---|---|
| Laboratory animals | NOD/SCID/IL2-/- (NSG) and NOD/Rag/Il2-/- (NRG) mice were used in this study. Housing was maintained in a 18–25 °C temperature range and 20–70% humidity range, with a 12 hour daylight cycle (on at 6:00am, off at 6:00pm). Surgery was carried out on female mice between the ages of 5–12 weeks. All experimental procedures were approved by the University of British Columbia Animal Care Committee |
| Wild animals | This study did not involve wild animals |
| Field-collected samples | This study did not involve samples collected from the field |
| Ethics oversight | The animal care committee of University of British Columbia approved all experimental procedures in this study |

Note that full information on the approval of the study protocol must also be provided in the manuscript.

# Human research participants

Policy information about studies involving human research participants

| | |
|---|---|
| Population characteristics | Age, gender (female), past and current diagnosis, hormone receptor status, past and current treatment, survival. Only survival was used in this manuscript. |
| Recruitment | Patients were recruited with informed consent during surgery or biopsy procedures. Patients were not selected for or excluded based on genomic information, so would not impact results. |
| Ethics oversight | approved by the Ethics Committees at the University of British Columbia |

Note that full information on the approval of the study protocol must also be provided in the manuscript.
