# Supplementary figures and images for: Single-cell genomic variation induced by mutational processes in cancer
Source: Nature. 2022 Oct 26;612(7938):106–15. doi: 10.1038/s41586-022-05249-0 (PMC9712114; doi:10.1038/s41586-022-05249-0)

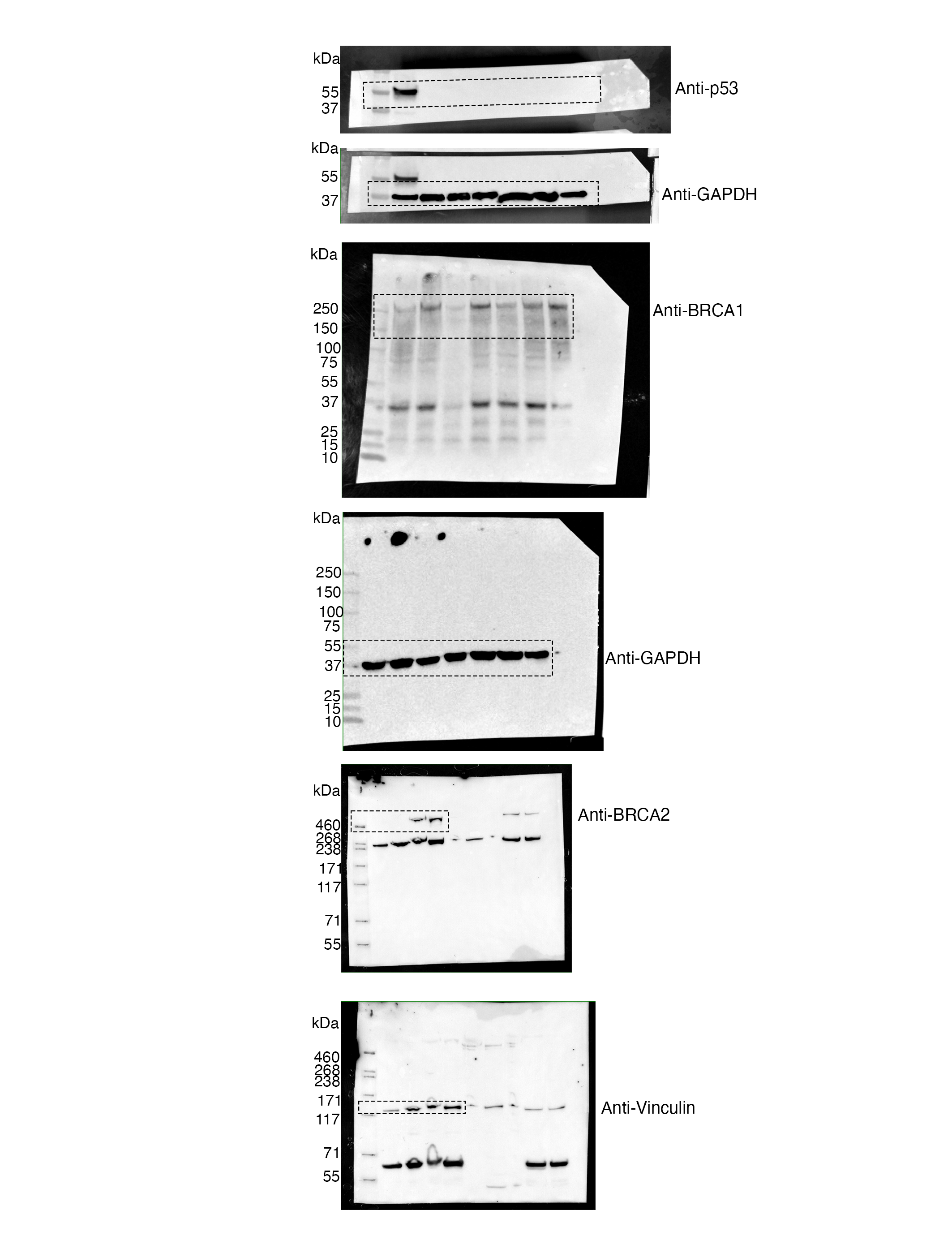

Supplement: Supplementary file 4 — - Full gel scans from Extended Figure 2. [file 41586_2022_5249_MOESM4_ESM.tif]
